# Supplementary figures and images for: A cost-effective and scalable barcoded library construction method for deep mutational scanning studies
Source: PLoS Biol. 2026 Feb 11;24(2):e3003645. doi: 10.1371/journal.pbio.3003645 (PMC12923136; doi:10.1371/journal.pbio.3003645)

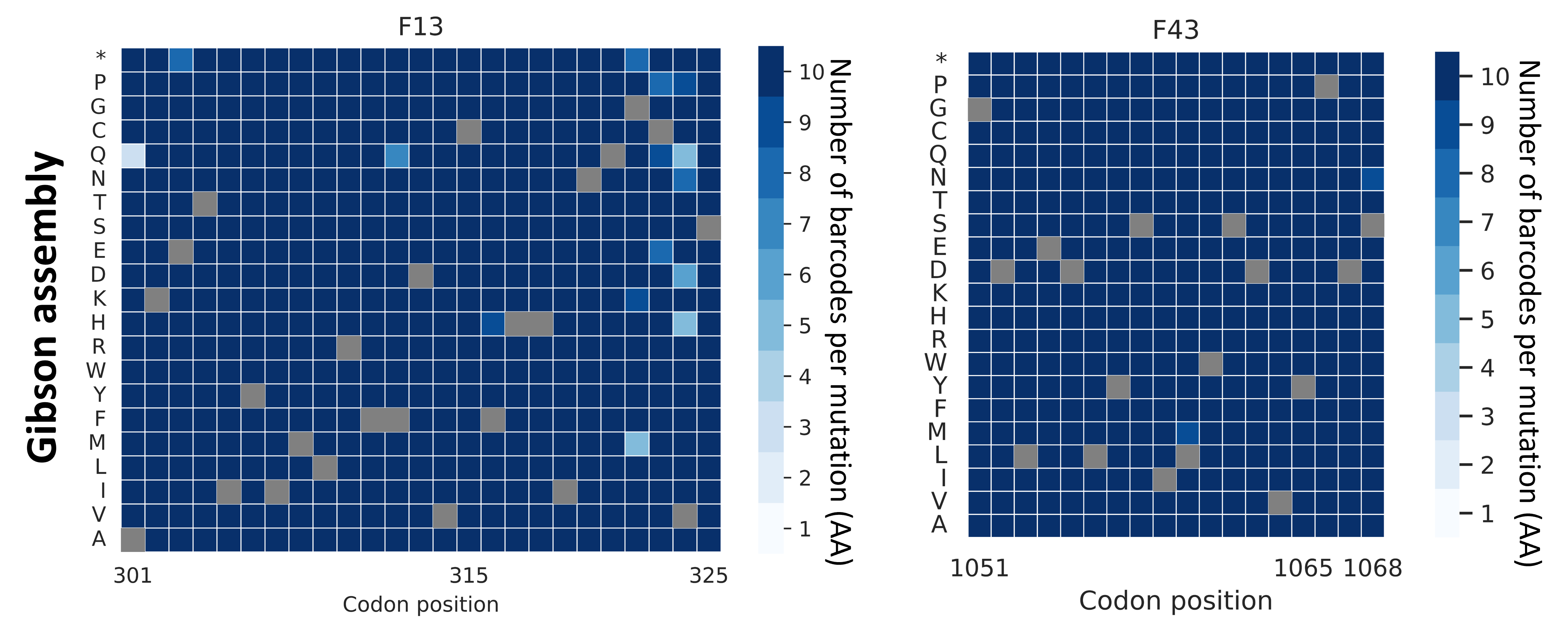

Supplement: S1 Fig — Heatmaps show the total number of unique barcodes for each possible amino acid substitution at each codon position in Pdr1 fragments F13 and F43. For each fragment, a total of 25,000 transformants were recovered and analyzed. Covered mutations are mutations represented by #barcodes >0, while barcode diversity represented by #barcodes >4 or >9 per mutation demonstrates an increasing number of replicates for a same mutation. Gray squares represent WT amino acids. In the heatmaps, the number of barcodes per mutation is clipped at 10. The numerical data underlying this graph is provided in S2 Data. (TIF) [file pbio.3003645.s001.tif]

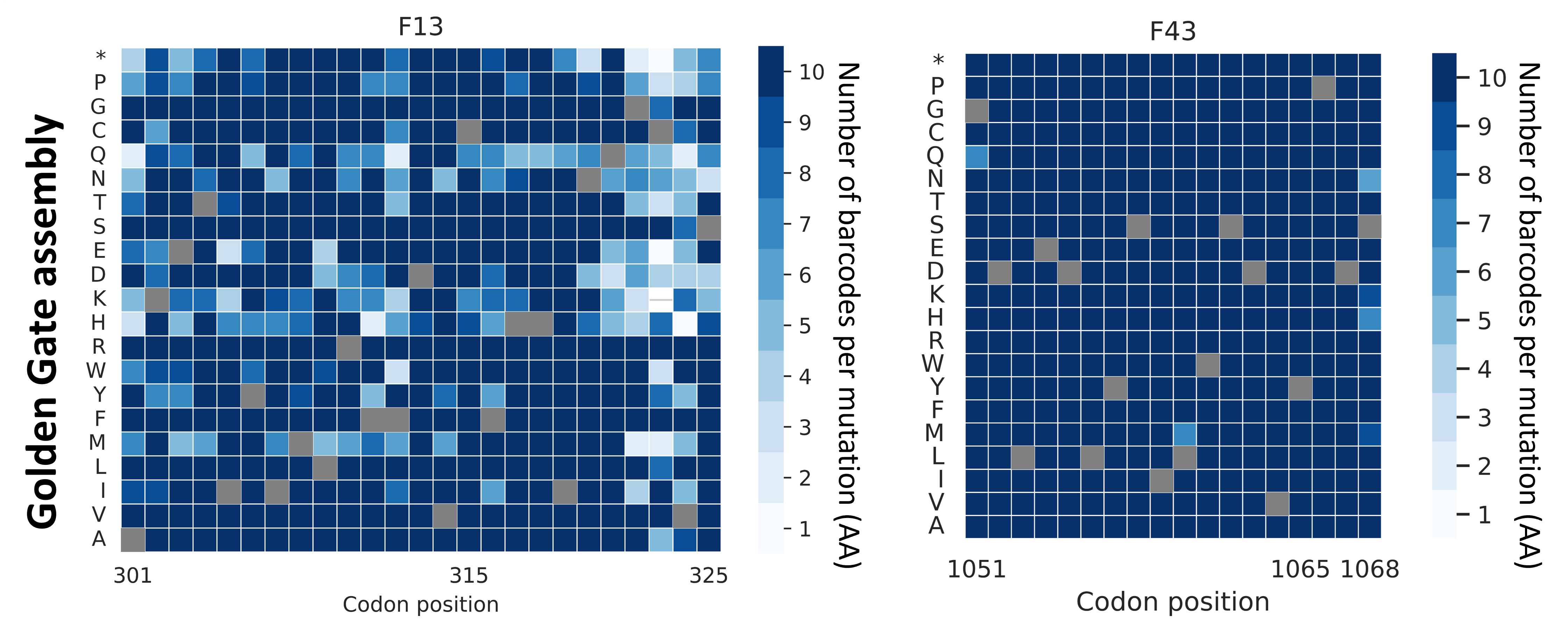

Supplement: S2 Fig — Heatmaps show the total number of unique barcodes for each possible amino acid substitution at each codon position in Pdr1 fragments F13 and F43. For each fragment, a total of 100,000 transformants were recovered and analyzed. Covered mutations are mutations represented by #barcodes >0, while barcode diversity represented by #barcodes >4 or >9 per mutation demonstrates an increasing number of replicates for a same mutation. Gray squares represent WT amino acids. In the heatmaps, the number of barcodes per mutation is clipped at 10. The numerical data underlying this graph is provided in S3 Data. (TIF) [file pbio.3003645.s002.tif]

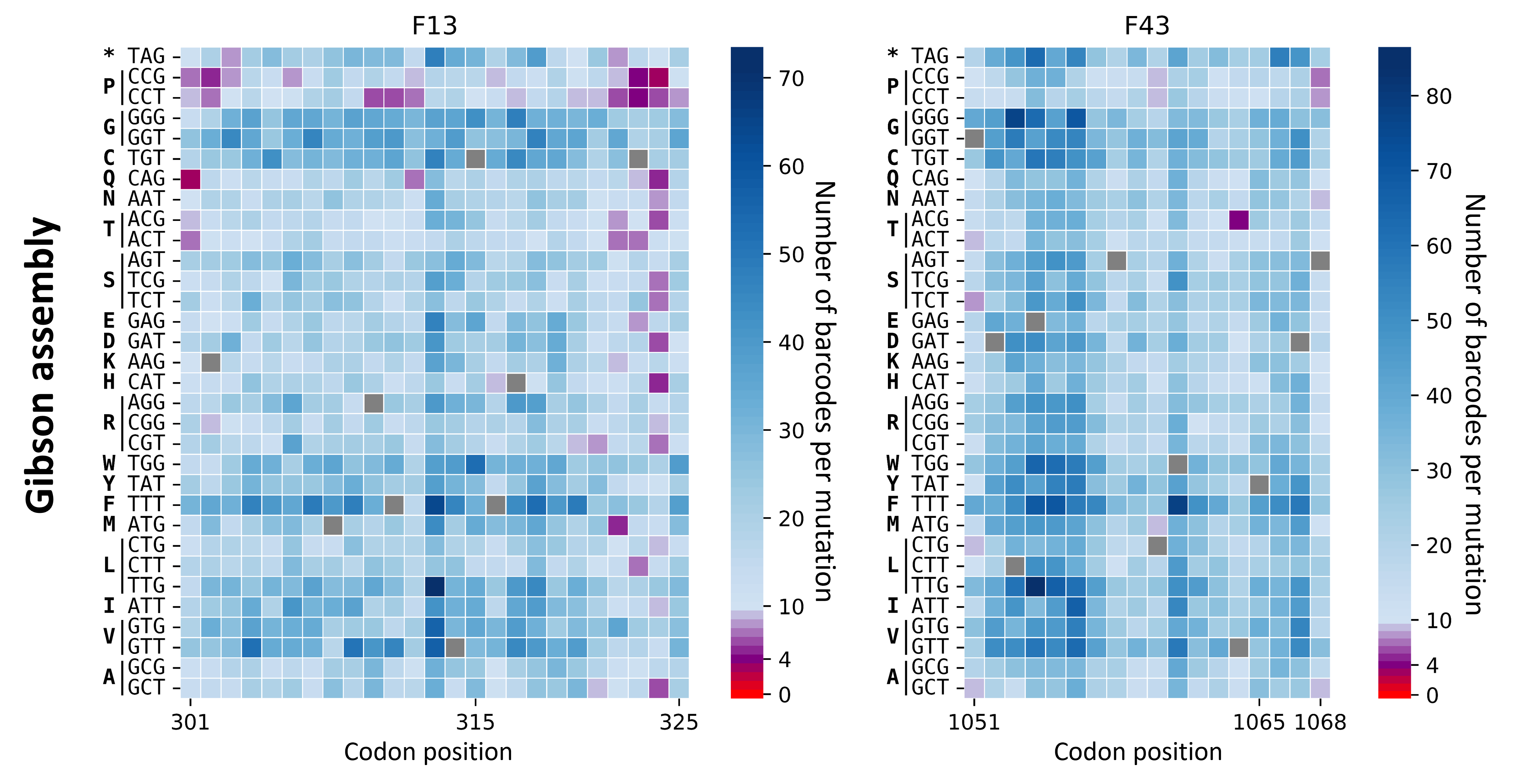

Supplement: S3 Fig — Heatmaps show barcode diversity for each possible NNK codon substitution at each codon position in Pdr1 fragments F13 and F43. For each fragment, a total of 25,000 transformants were recovered and analyzed. Barcode diversity is shown using an unclipped color scale, allowing visualization of the full range of barcode counts. Mutations covered by high barcode diversity (#barcodes ≥10 and ≥4) are represented by a blue and purple scale, respectively, while lower barcode diversity (#barcodes <4) is represented by a red scale. Gray squares represent WT amino acids. The numerical data underlying this graph is provided in S2 Data. (TIF) [file pbio.3003645.s003.tif]

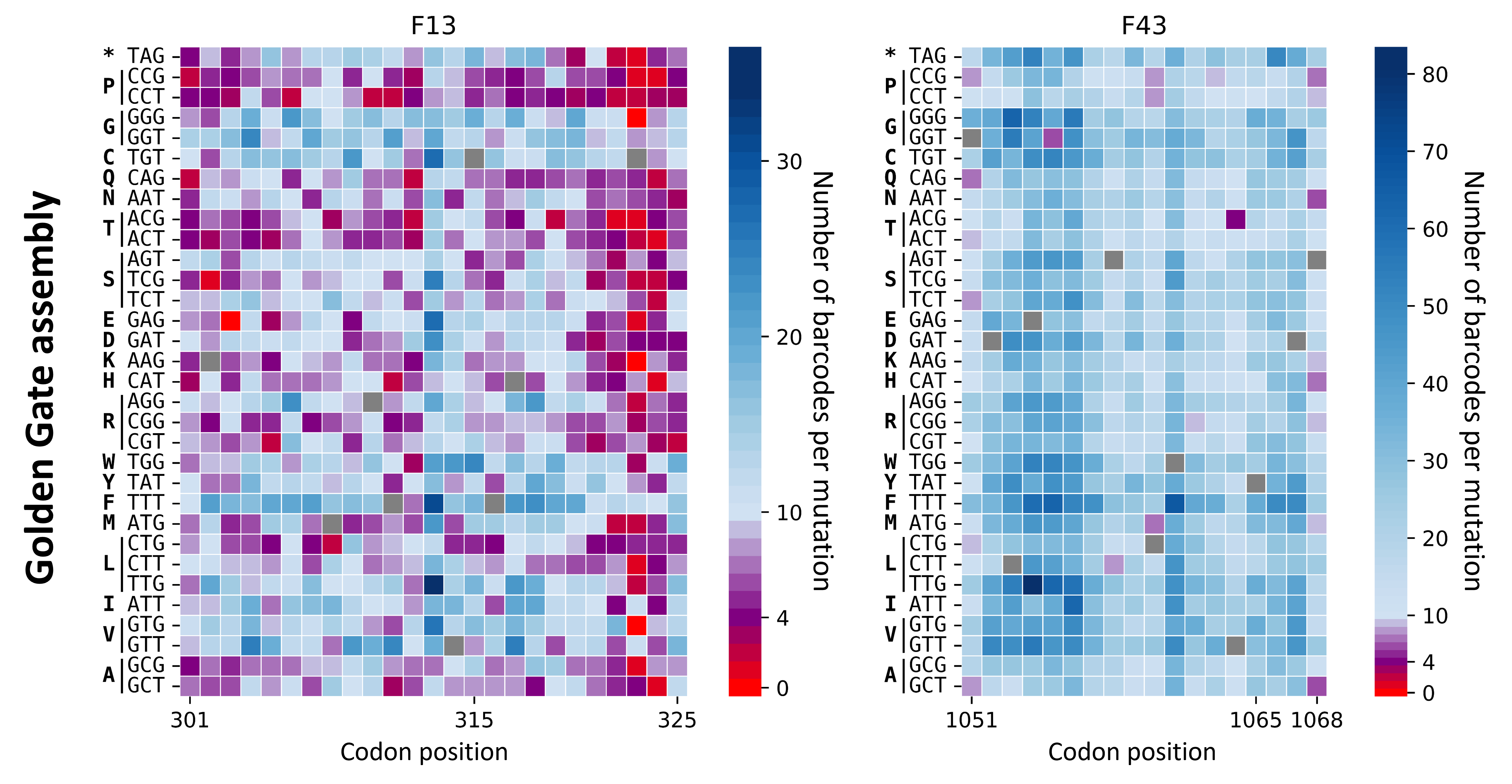

Supplement: S4 Fig — Heatmaps show barcode diversity for each possible NNK codon substitution at each codon position in Pdr1 fragments F13 and F43. For each fragment, a total of 100,000 transformants were recovered and analyzed. Barcode diversity is shown using an unclipped color scale, allowing visualization of the full range of barcode counts. Mutations covered by high barcode diversity (#barcodes ≥10 and ≥4) are represented by a blue and purple scale, respectively, while lower barcode diversity (#barcodes <4) is represented by a red scale. Gray squares represent WT amino acids. The numerical data underlying this graph is provided in S3 Data. (TIF) [file pbio.3003645.s004.tif]

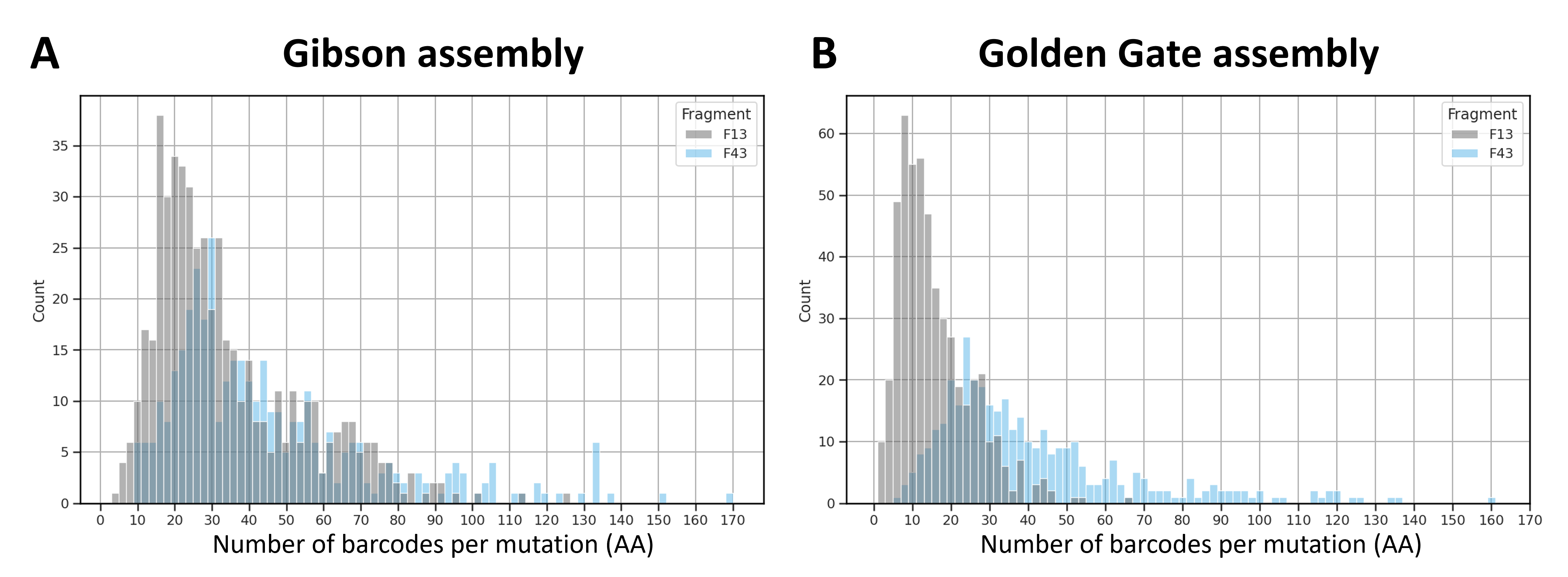

Supplement: S9 Fig — Histograms show the number of unique barcodes associated with each amino‑acid substitution in fragments F13 (gray) and F43 (blue). For each fragment, the x‑axis indicates the number of barcodes linked to a given amino‑acid substitution, and the y‑axis shows the number of substitutions observed at each barcode count. The numerical data underlying these graphs is provided in S2 and S3 Data. (TIF) [file pbio.3003645.s009.tif]

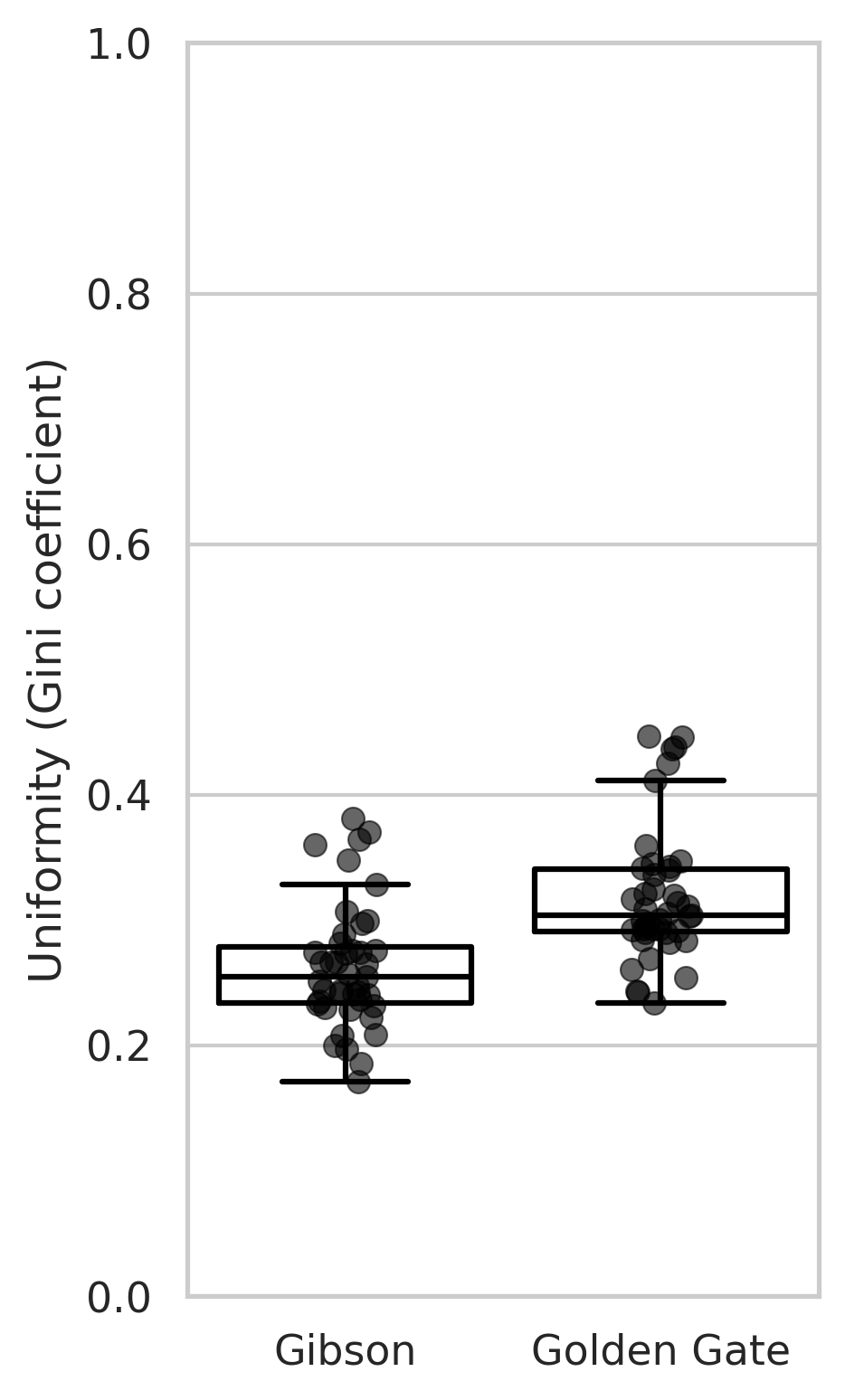

Supplement: S10 Fig — Boxplots represent the distribution of Gini coefficients (ranging from 0 to 1) calculated for each fragment, where lower values indicate more uniform coverage and higher values indicate increased inequality in representation. The numerical data underlying this graph is provided in S4 Data. (TIF) [file pbio.3003645.s010.tif]

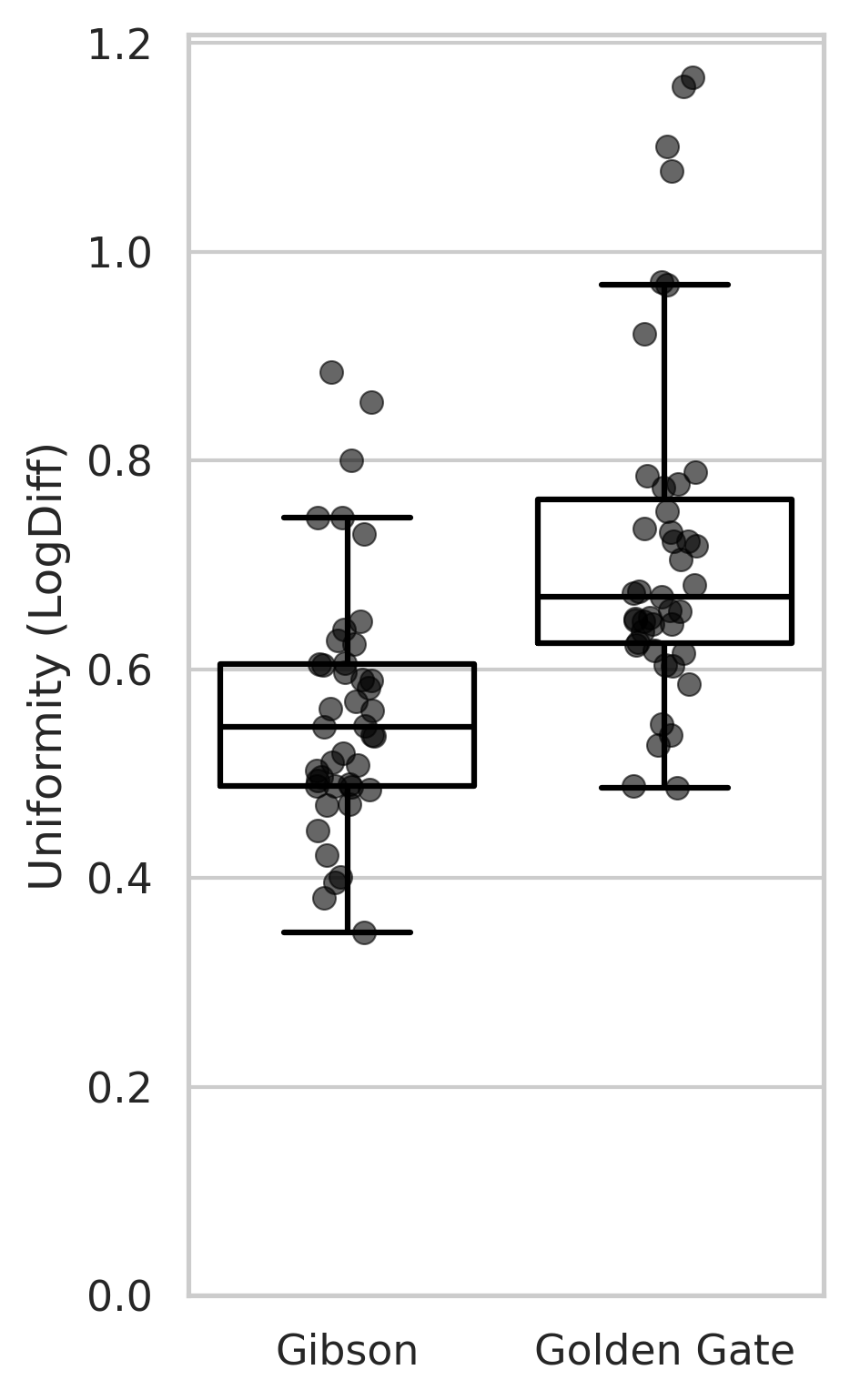

Supplement: S11 Fig — Boxplots show the distribution of uniformity scores for each fragment, defined as the log difference between the 90th and 10th percentiles of mutant read counts. Lower scores indicate more uniform representation, while higher scores indicate greater inequality. The numerical data underlying this graph is provided in S4 Data. (TIF) [file pbio.3003645.s011.tif]

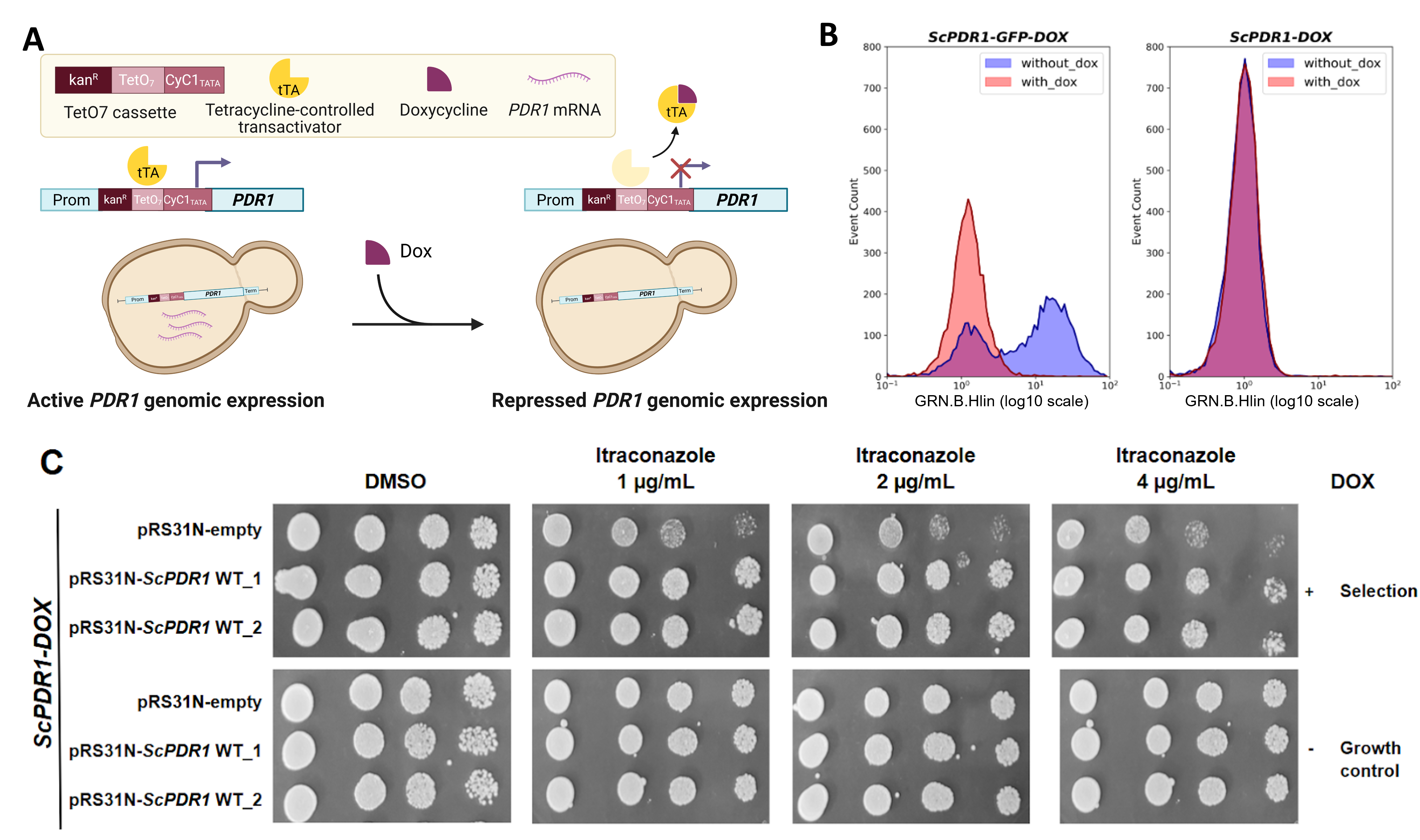

Supplement: S12 Fig — A) Inducible control of genomic PDR1 expression in Saccharomyces cerevisiae using a doxycycline-repressible promoter (TetO7). Created in BioRender. Barff, T. (2025) https://BioRender.com/zo8wlgo. B) Validation of genomic PDR1 repression in the ScPDR1-DOX strain. PDR1 expression level was assessed by flow cytometry using a GFP fusion as a reporter. Fluorescence intensity is shown for the strain expressing GFP (left panel: ScPDR1-GFP-DOX) and the negative control strain without GFP (right panel: ScPDR1-DOX) in media with (red) or without (blue) DOX. GFP intensity was analyzed on the main population of morphologically normal cells (FSC-H<15000 and FSC-A<15000). 5,000 events were recorded per replicate. Three independent biological replicates were analyzed per condition. The numerical data underlying this graph is provided in S8 Data. C) in ScPDR1-DOX by plasmid-expression of ScPDR1 (pRS31N-ScPDR1 WT) along with the control strain (pRS31N-empty). Growth conditions: DMSO (control) or Itraconazole (ITR) (antifungal). (TIF) [file pbio.3003645.s012.tif]

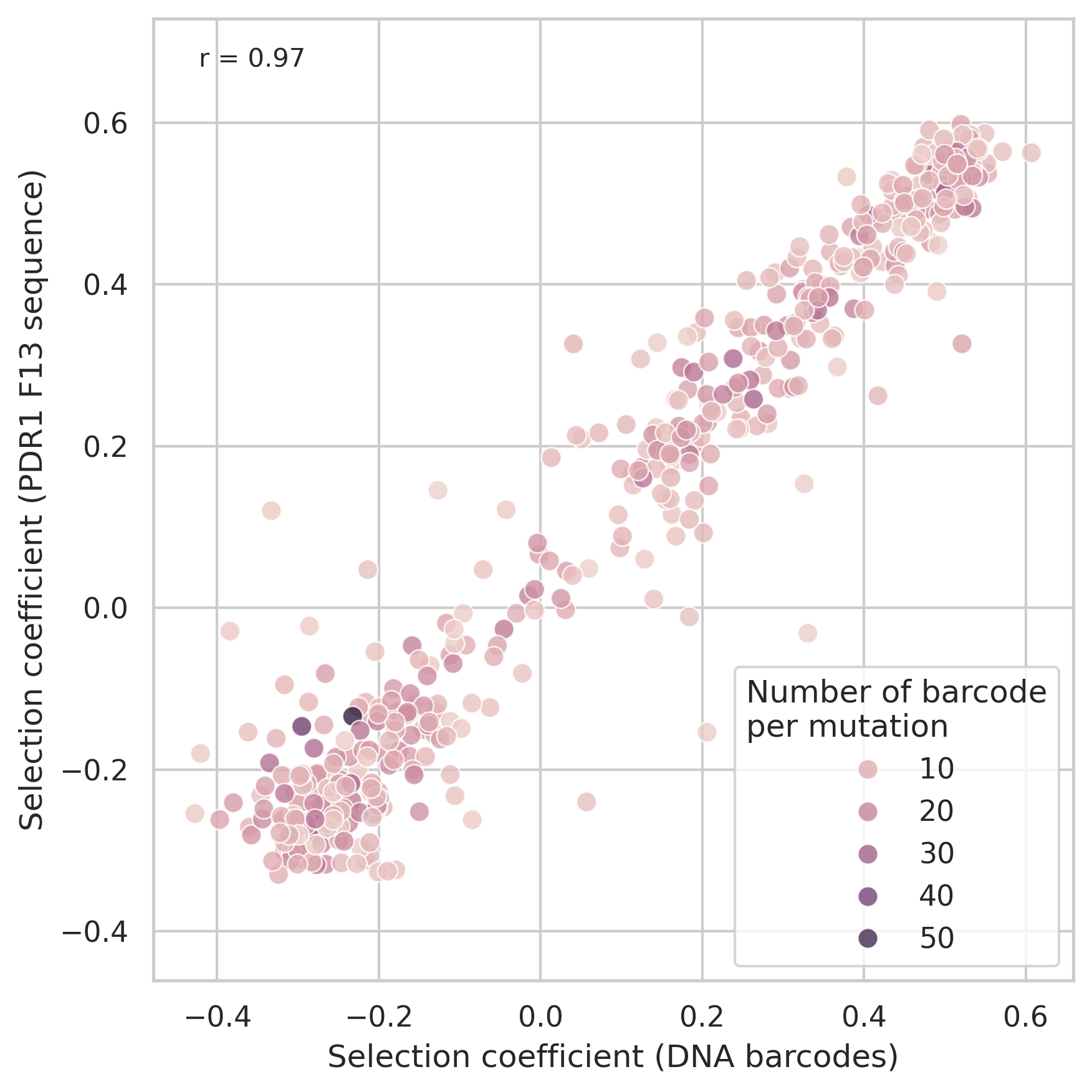

Supplement: S13 Fig — The strong correlation (Pearson r = 0.97) indicates that barcode-based estimates accurately recapitulate the directly measured fitness effects. The number of barcodes per mutation does not affect the correlation. The numerical data underlying this graph is provided in S5 and S6 Data. (TIF) [file pbio.3003645.s013.tif]

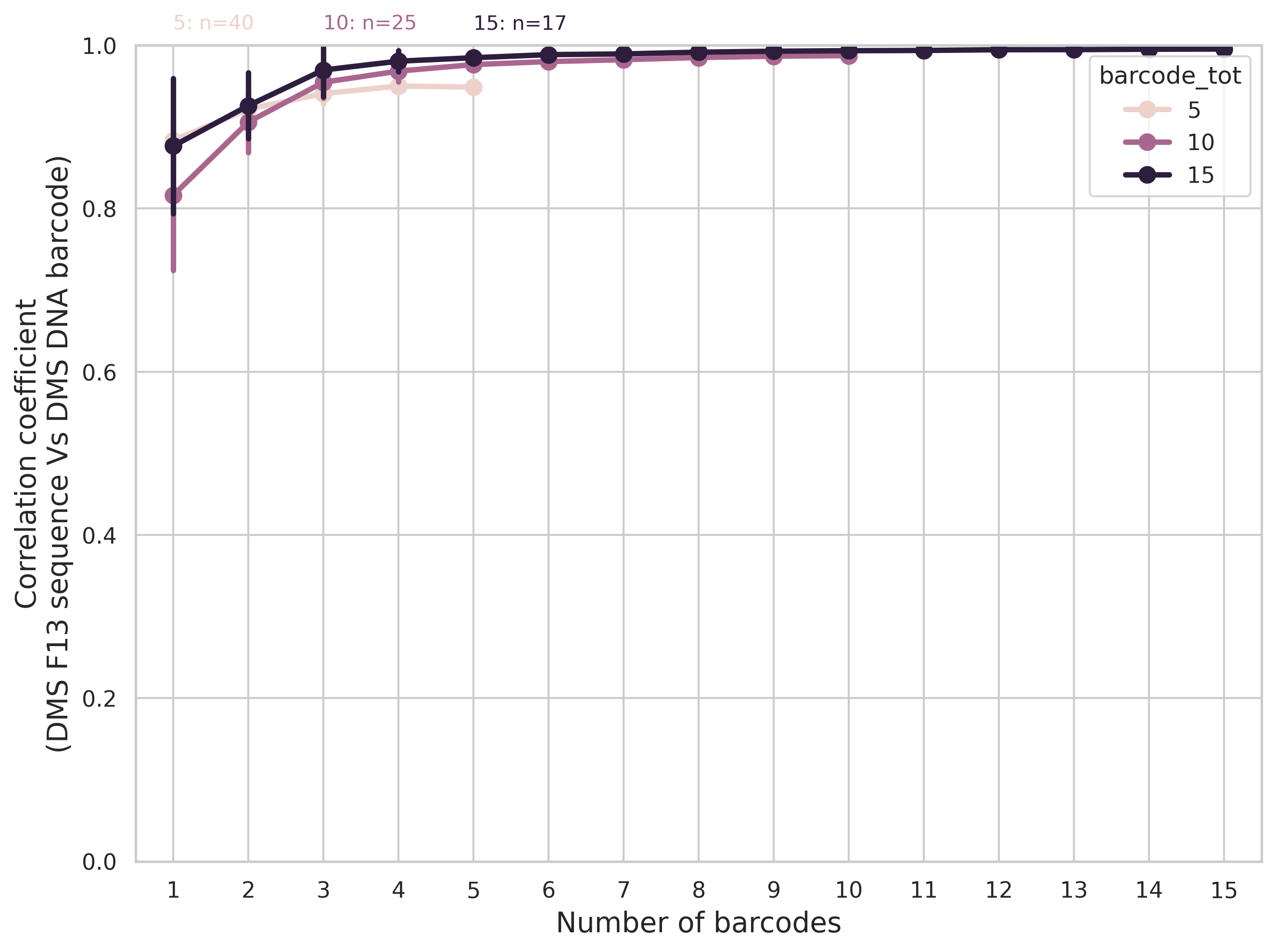

Supplement: S14 Fig — Curves correspond to variants with exactly 5 (n = 40), 10 (n = 25), or 15 (n = 17) barcodes. Means and confidence intervals were calculated from 100 random barcodes subsamplings per variant. The high correlation observed even for variants with only five barcodes indicates that the overall correlation is not driven only by highly diversified variants. The numerical data underlying this graph is provided in S9 Data. (TIF) [file pbio.3003645.s014.tif]
