# Supplementary material for: A cost-effective and scalable barcoded library construction method for deep mutational scanning studies
Source: PLoS Biol. 2026 Feb 11;24(2):e3003645. doi: 10.1371/journal.pbio.3003645 (PMC12923136; doi:10.1371/journal.pbio.3003645)
Supplement: Attachment — Submitted filename: Response to Reviewers.docx [file pbio.3003645.s036.docx]

Onglet 1

**Peer Review Information**

**Journal:** PLOS Biology as a *Methods and Resources Article*

**Manuscript Title:** Making deep mutational scanning accessible: a cost-efficient approach to construct barcoded libraries for genes of any length

**Corresponding author name:** Christian Landry

**Authors responses:**

We would like to thank all the reviewers for taking the time to read our manuscript and highlighting how it can be improved. We are very grateful for their evaluation of our work. In this document, we attempt to address all the concerns and questions raised by the reviewers. The reviewers' comments and questions are in bold and underlined, and our responses are given in blue below each comment. For each reviewer, general comments are followed by numbered comments. All line numbers refer to the revised manuscript, and passages highlighted in yellow are additions to the manuscript.

# Reviewer #1: Jann et al. present a method for constructing saturation mutagenesis libraries coupled to nucleic acid barcodes. They demonstrate the efficacy of this method by performing construction of two barcoded NNK tiles (one 75-nt, one 54-nt - (Tile 43, Supporting Tables)), and performing a deep mutational scanning experiment on one 75-nt tile of the PDR1 sequence. This motivation behind this method is the need for cost-effective deep mutational experiments of full-length genes (for example, the coding sequence for PDR1 is 3204 bp). Two major strategies exist - one can either mutate each tile separately and perform separate selections and then sequencing ('tile-seq'). Alternatively, one can couple a nucleic acid barcode to a specific variant of interest and sequence the barcode (for an early example see Sarkisyan, Karen S., et al. "Local fitness landscape of the green fluorescent protein." Nature 533.7603 (2016): 397-401.). The Sarkisyan paper used a molecular biology 'hack' to connect the barcode to a variant using short read sequencing; there are other - conceptually similar - short read coupling strategies as well (Petersen …Whitehead Nat Comms 2024). Alternatively, there are methods to pre-assign a barcode to a gene variant, avoiding barcode-variant haplotyping altogether (Ranganathan Nat Comms 2019). More commonly, the barcode is coupled to the variant of interest using long read sequencing by PacBio or Oxford Nanopore. The disadvantage of the long read strategy is the sequencing cost and throughput. In the author's strategy, they use a two-step method where a barcode is covalently fused to a 75-nt gene tile. In step 1, a Gibson assembly is performed. In step 2, the full plasmid is reconstituted via Golden Gate assembly using the 3' portion of the remaining gene. The advantage of the Jann method is resolving the barcode to the gene variant after step 1 via short read sequencing. The major disadvantage of their method is in the need for n separate long-length PCRs to generate the linearized DNA for Gibson assembly in step 1 (here n is the number of tiles). The paper flows well and has well-described and executed experiments. However - as described below - it is unclear on reading whether this method represents an improvement over current methods for scanning long full-length genes. A more thorough evaluation and comparison to the current literature, as well as additional analysis would improve the quality of this contribution.

We sincerely thank the reviewer for the time and effort dedicated to evaluating our manuscript and for the constructive comments provided. These suggestions have helped us improve the clarity, precision, and contextualization of our work. We have carefully revised the manuscript accordingly and expanded several sections (Main text, Method, and Supplementary Material) to better highlight the distinguishing features, strengths, and limitations of our two-step barcoded DMS library construction strategy. We hope that the revisions and additional analyses now fully address the reviewer’s concerns and expectations.

**Major concerns**

## Comment #1.1. The method is specifically designed to address mutagenesis of very large genes. However, the paper does not appreciably contextualizing similarities/differences between this method and previous methods, and the introduction does not place the present work with the current literature on technical improvements for large genes. For example, it appears that the method presented here is very similar to the method employed by Sri Kosuri et al in two papers, one of which is cited by the authors of the present manuscript: [Jones et al., 2020, eLife] and [Howard et al., 2025, eLife]. The authors should specify if indeed the method is the same, or if not, how it differs. What are the distinguishing features? What are the strengths of this short read haplotyping strategy vs. other approaches that exist in the literature? Apt comparisons would be with nicking mutagenesis (Mighell et al., 2023, PLoS One) or DIMPLE (Macdonald et al., 2023, Genome Biology). These methods are much more widely used, can incorporate NNK encoded nucleotides, and are alternatives for generating the library when coupled with barcodes and long read haplotyping.

Response:

We thank the reviewer for this critical comment. Unfortunately, the short format of our initial manuscript did not allow us to make these comparisons. We have added this segment to the discussion where we discuss the similarities and differences with other methods.

Main text, *started line 159*:

## Comment #1.2. The authors list 3 parameters key for satmut libraries: informative barcodes, mutation coverage, and barcode diversity. However, one parameter is missing from this list: uniformity of variants. This is important for limiting sequencing costs: two libraries with the same percent of informative barcodes, mutation coverage, and barcode diversity could have very different uniformity, leading to more sequencing reads required to assess the less uniform one. Metrics exist for evaluating uniformity in tiled gene libraries (e.g., Mighell et al., 2023, PLoS One), and would encourage an analysis of tile 13 and 43 libraries using this or similar metrics.

Response:

We thank the reviewer for this important suggestion and totally agree that variant uniformity is a key parameter that complements informative barcodes, mutation coverage, and barcode diversity in evaluating the quality of DMS libraries. To address this point, we have now quantified library uniformity using two different metrics:

1. The Gini coefficient, a well-established measure of distribution bias previously applied to mutational libraries [[1]](https://paperpile.com/c/Ryldux/LxcX). The Gini coefficient ranges from 0 to 1, where 0 indicates perfect uniformity (equal representation of all variants) and higher values indicate increasing bias.
2. The uniformity score proposed by Mighell et al. [[2]](https://paperpile.com/c/Ryldux/mJgg), defined as the log difference between the 90th and 10th percentiles of mutant read counts per fragment. This metric captures the spread of variant abundance across the library, with smaller values reflecting higher uniformity.

We computed both metrics for the complete *PDR1* plasmid library after each cloning step (Gibson and Golden Gate assemblies) and for the initial T₀ pool used in the DMS experiment to assess uniformity before selection. The results (presented in Supplementary Figures 3 and 4 and summarized in Supplementary Table 8) show that the Gini coefficients and uniformity scores remain low, confirming a good degree of uniformity across fragments. As expected, Golden Gate assemblies showed a slight increase in Gini coefficient relative to Gibson assemblies, reflecting minor bottlenecks associated with multi-step cloning, but the overall uniformity remains good across all 43 fragments (always < 0.5).

Supplementary Figure 3 and 4:

**Supplementary Figure 3: Assessment of library uniformity after each cloning step (Gibson and Golden Gate assemblies) using the Gini coefficient** [[1]](https://paperpile.com/c/Ryldux/LxcX)**.** Boxplots represent the distribution of Gini coefficients (ranging from 0 to 1) calculated for each fragment, where lower values indicate more uniform coverage and higher values indicate increased inequality in representation.

**Supplementary Figure 4: Assessment of library uniformity after each cloning step (Gibson and Golden Gate assemblies) using the uniformity score** [[2]](https://paperpile.com/c/Ryldux/mJgg)**.** Boxplots show the distribution of uniformity scores for each fragment, defined as the log difference between the 90th and 10th percentiles of mutant read counts. Lower scores indicate more uniform representation, while higher scores indicate greater inequality.

Mentioned in main text, *started line 190*:

Mentioned in method, *started line 530*:

For additional context, we also computed both metrics for a previously published DMS plasmid library generated from our laboratory using a standard one-by-one cloning approach (*CaERG11* library [[3]](https://paperpile.com/c/Ryldux/uDId)). The *CaERG11* library was constructed using a highly precise, but low-throughput, position-by-position cloning strategy. DNA fragments synthesized by Twist Bioscience were cloned codon-by-codon into a yeast expression vector, and the individual codon libraries were then pooled together proportionally based on the number of mutants. This meticulous process allows to achieve a near-perfect uniformity of variant representation, which can serve here as a reference. Comparison between the two datasets (Supplementary Table 7) indicates that the uniformity of our barcoded strategy is certainly less efficient but it is perfectly acceptable for robust DMS experiments (Gini coefficient: 0.52). Importantly, our strategy remains more cost-effective and scalable.

Supplementary Table 7:

Together, these analyses strengthen the manuscript by demonstrating that our construction strategy maintains acceptable uniformity of variant representation, ensuring that sequencing depth can be efficiently used across all variants.

## Comment #1.3. My suggestion is to remove the cost comparison or to greatly strengthen the rigor behind the claims (Supp Fig 1; Table S1). First, the synthesis argument obscures the relevant difference between sat mutagenesis & barcoded oPools. No one outside of a handful of academic labs and in industry would consider gene synthesis at scale like this. Second, the cost comparison presented here omits time and labor requirements of the method, which appear to be quite substantial. Specifically, the example gene PDR1 requires 43 sub-libraries to be constructed, each of which requires long-range PCR amplification of the original wild-type vector. These PCRs can be very tricky and often require multiple rounds of optimization of conditions. 43 of these reactions is quite a molecular biology load, and would scale unfavorably as you increase the length of YFG. Third, the claims behind the numbers are not well sourced and cannot be independently verified. In my opinion, the quality of the paper does not rest on an economic argument. In such cases, it's best to remove a cost comparison.

Response:

Following the reviewer’s suggestion we have now removed the cost comparison.

About the labor time required, we have now completed the construction of all 43 sub-libraries of the *PDR1* gene. This allows us to present a detailed experimental timeline for constructing the full *PDR1* library (Supplementary Table 9), providing an overview of the expected time frame for each step. While this two-step strategy is more time-consuming than standard approach, it is important to note that cloning step is still required even with commercial library synthesis approaches.

In our experience, the long-range PCRs required for plasmid backbone amplification were manageable and reproducible using high-fidelity polymerases and cycling conditions presented. The complete set of 43 backbones and corresponding oPools was prepared within about 1–2 weeks, indicating that this step is relatively straightforward in practice. We believe that the point raised by the reviewer further demonstrates the importance of having well documented and developed methods such as what we are doing here in this manuscript. We hope that the detailed protocol provided will facilitate similar results in other laboratories.

Finally, regarding completely removing the cost comparison, even with the changes made, we will leave it up to the publisher to decide.

## Comment #1.4. What is claimed in the paper (a method for size independent barcoded sat mut library generation) vs. what is demonstrated (barcoded libraries for two short tiles) is not reflected in the title, abstract, or introduction. Accurate description of what is demonstrated in the method, along with a discussion of strengths and limitations (n PCRs, n synthesized gene fragments of YFG, n parallel Gibson Assemblies, n parallel Golden Gate assemblies) in the discussion would improve the readability of the manuscript. Specifically, the authors did not demonstrate the method on multiple genes; did not create a barcoded full length saturation NNK library of their gene of interest; and did not perform a deep mutational scan on the full-length gene. While I don't think these experiments are necessary for publication of the method, they are necessary if the abstract and title remain unchanged.

Response:

We agree that the title, abstract, and introduction should accurately reflect the scope of what is experimentally demonstrated in the present work. Our main goal in this manuscript is to describe and validate a straightforward and cost-effective barcoding strategy for constructing DMS plasmid libraries for genes of any length, rather than to present a full-length functional screen.

Some of the steps mentioned by the reviewer, such as phenotypic screening of the complete mutant library, are essential components of a DMS workflow but are common to all DMS experiments and do not constitute a methodological innovation. For this reason, we chose not to include them here and will present the large-scale functional screening results in a dedicated follow-up manuscript focused on the biological outcomes of *PDR1* variants.

Regarding the demonstration of scalability, we would like to clarify that we have now constructed the complete plasmid library for the entire *PDR1* gene (43 fragments, 1,068 amino acids), in addition to the two representative fragments (F13 and F43) presented in the main text. The construction metrics for each sub-library (number of transformants, informative barcode counts, mutation coverage, barcode diversity, and Gini coefficients) are now provided in Supplementary Table 8 and visually summarized in Supplementary Figures 7 and 8. Furthermore, we include an estimated experimental timeline for constructing the full 43-fragment library in Supplementary Table 8 to give readers a realistic view of the experimental time involved.

Supplementary Figure 7

**Supplementary Figure 7: Library quality control after Gibson assembly of the full-length *PDR1* sequence (43 fragments).** Heatmap displays the barcode diversity associated with each possible amino acid substitution at each codon position, with values clipped at 10 barcodes per mutation for clarity.

Supplementary Figure 8

**Supplementary Figure 8: Library quality control after Golden Gate assembly of the full-length *PDR1* sequence (43 fragments).** Heatmap displays the barcode diversity associated with each possible amino acid substitution at each codon position, with values clipped at 10 barcodes per mutation for clarity.

Manuscript, *started line 553*:

We agree that the present study does not report (i) the generation of barcoded saturation libraries for multiple unrelated genes or (ii) a pooled functional screen of the full-length *PDR1* gene (43 sub-librairies simultaneously). However, the successful construction and validation of the full-length *PDR1* mutant library, together with the high performance demonstrated for the functional screen (correlation between *PDR1* F13 sequence VS barcode and correspondence with data from literature), provide strong evidence of the main goal described in our manuscript.

Finally, in response to the reviewer’s suggestion, we have added more details in the methodology section including the number of Gibson or Golden Gate reactions per fragment library, the amount or the number of transformations for each cloning step.

Methods section, *started line 446*:

Methods section, *started line 463*:

This additional information should make the protocol more transparent and reproducible for the community. We believe that all the additional information of the revised manuscript (Supplementary Table 8 and 9, as well as Supplementary Figure 7 and 8), strongly support the scalability and applicability of our approach to genes of any size.

**Smaller comments**

## Comment #1.5. Figure 1C and 1D: it appears that the number of barcodes per variant is clipped at 10. This should either be mentioned explicitly, or better the raw values should be shown (this is better because the reader would get a better sense of the uniformity of the generated libraries).

Response:

We thank the reviewer for this suggestion. We have now made it explicit in the figure legend that the number of barcodes per mutation in Figure 1C and 1D is clipped at 10. In addition, we updated the heatmaps to use a discretized color scale, which makes it easier to visually distinguish the number of barcodes associated with each mutation. Because the main goal of this figure is to demonstrate that most mutations are represented by >4 barcodes, the minimum threshold we determined necessary for robust correlation, we used a scale optimized to highlight barcode counts from 0 to 10. This allows readers to focus on variants with lower barcode diversity, which are the most critical for assessing library quality.

Figure 1 - C and D:

Legend of Figure 1 - C and D, *started line 119*:

In addition, to improve transparency and address the reviewer’s comment, we have included unclipped heatmaps showing the full range of barcode counts in Supplementary Figures 1 and 2.

Supplementary Figure 1:

**Supplementary Figure 1: Barcode diversity after Gibson assembly.** Heatmaps show the total number of unique barcodes for each possible amino acid substitution at each codon position in *PDR1* fragments F13 and F43. For each fragment, a total of 25,000 transformants were recovered and analyzed. Some amino acids, such as serine, leucine, and arginine, are more accessible with the NNK library (six codons each instead of four) and thus show higher diversity. Grey squares indicate wild-type (WT) amino acids.

Supplementary Figure 2:

**Supplementary Figure 2: Barcode diversity after Golden Gate assembly.** Heatmaps show the total number of unique barcodes for each possible amino acid substitution at each codon position in *PDR1* fragments F13 and F43. For each fragment, a total of 100,000 transformants were recovered and analyzed. Some amino acids, such as serine, leucine, and arginine, are more accessible with the NNK library (six codons each instead of four) and thus show higher diversity. Grey squares indicate wild-type (WT) amino acids.

## Comment #1.6. Figure 2C: when evaluating the effect of number of barcodes on replicate correlation; was the number of short-read sequencing reads accounted for? Variants with higher number of barcodes will also tend to be more abundant in the library and their fitness will be better estimated simply due to increase short read coverage.

Response:

We thank the reviewer for raising this point. We agree that variants associated with a higher number of barcodes may also tend to improve the precision of fitness estimates and have an influence on the correlation coefficients. To address this point, we reanalyzed our data by stratifying data into categories based on their barcode representation and then recalculating the selection coefficient correlations (*PDR1* F13 sequence VS DNA barcodes) within each category (Supplementary Figure 9).

This analysis revealed that even variants associated with only five barcodes exhibited a strong selection coefficient correlation between DMS from *PDR1* F13 sequences and from barcodes, demonstrating that our barcode strategy is robust and not only driven by the variant with higher number of barcodes.

Supplementary Figure 9:

**Supplementary Figure 9: Correlation between the selection coefficients measured with direct sequencing of the mutated F13 coding region and DNA-barcode inference, as a function of the number of barcodes per variant considered.** Curves correspond to variants with exactly 5 (n = 40), 10 (n=25), or 15 (n=17) barcodes. Means and confidence intervals were calculated from 100 random barcodes subsamplings per variant. The high correlation observed even for variants with only five barcodes indicates that the overall correlation is not driven only by highly diversified variants.

Mentioned in the main text, *started line 750*:

Furthermore, as expected, we observed a positive correlation between the number of barcodes per variant and the number of sequencing reads per variant (see following Figure A), confirming that barcode abundance contributes to read coverage. However, despite this correlation, the strong agreement between barcode-based and direct *PDR1* F13 sequencing–based fitness estimates remained consistent even in low-barcode categories, supporting the reliability of our approach and the robustness of the observed correlations.

Figure A:

**Figure A: Relationship between barcode diversity and sequencing depth.** Read counts per mutation increase with the number of barcodes associated with that mutation. Colors denote the different *PDR1* fragments.

## Comment #1.7. Figure 2 - what is the internal reproducibility of the assay (barcode vs. barcode or tile vs. tile)? And, how does this compare with the Figure 2 graph shown comparing barcode vs. tile?

Response:

We thank the reviewer for raising this point regarding the internal reproducibility of the assay. To address this, we evaluated reproducibility at several levels of correlation within experiments.

Within the barcoded library, we quantified barcode-level reproducibility by calculating pairwise correlations among barcodes corresponding to the same variant. The median pearson correlation was 0.70 (interquartile range: 0.59 - 0.75), indicating good overall consistency despite some expected barcode-level noise. Aggregating barcode data improved reproducibility at the codon level, with a mean correlation of 0.93 across codons encoding the same amino acid (Figure B).

Figure B:

**Figure B: Pairwise comparisons of selection coefficients among synonymous codons for the barcodes library assay.** The lower triangle shows scatter plots of codon-level selection coefficients for codons encoding the same amino acid, and the upper triangle reports the corresponding Pearson correlation coefficients.

For the *PDR1* F13 sequencing-based assay, correlations among synonymous codons were similarly high (mean *r* = 0.95) (Figure C). Finally, reproducibility between independent replicate *PDR1* F13 sequencing experiments was high (mean *r* = 0.98), confirming the robustness of the assay across biological replicates.

Figure C:

**Figure C: Pairwise comparisons of selection coefficients among synonymous codons for the *PDR1* F13 sequencing-based assay.** The lower triangle shows scatter plots of codon-level selection coefficients for codons encoding the same amino acid, and the upper triangle reports the corresponding Pearson correlation coefficients.

Finally, comparison between the DNA barcode and F13 sequence assays yielded a correlation of 0.97 (Supplementary Figure 6), indicating that measurements are highly consistent across experimental designs and that the residual noise observed at the barcode level does not substantially affect the overall signal.

Supplementary Figure 6:

**Supplementary Figure 6: Correlation between the selection coefficients measured with direct sequencing of the mutated *PDR1* F13 coding region and DNA-barcode inference, using all usable barcodes.** The strong correlation (Pearson *r* = 0.97) indicates that barcode-based estimates accurately recapitulate the directly measured fitness effects. The number of barcodes per mutation does not affect the correlation.

## Comment #1.8. The barcoding scheme uses position specific sequences along with degenerate sequences to tag each variant at a given position. Do the authors have any evidence that this scheme leads to higher fidelity variant-barcode associations compared with fully random barcodes?

Response:

We thank the reviewer for this comment. We have not compared our strategy with a strategy where a purely random sequence would be added to the variant sequence. This design choice is mainly due to the current oligonucleotide synthesis constraints: commercially available cost-effective oPools allow a maximum of nine degenerate nucleotides per oligonucleotide. Three of which are required for the NNK codon, leaving six for barcode diversity. Including a short fixed sequence specific to each codon position allowed to remain within synthesis limits and does not prevent multiple barcodes per mutation, a key requirement for internal replication and statistical robustness in downstream analyses. We also thought that having a barcode specific for each fragment and codon could help if cases of errors occur whereby diversity is missing from a single codon or a fragment, which would be easily spotted from the representation of the codon and fragment-specific barcodes and their associated variants.

# Reviewer #2 (Olivier Tenaillon, signs review): In this manuscript by Jessica Jann entitled "Making deep mutational scanning accessible: a cost-efficient approach to construct barcoded libraries for genes of any length" propose a cost efficient method to do DMS in long genes with an coupling with Barcode.

**The method is astute and straight forward and propose an intermediate coupling with barcode, which is truly relevant, as altenrative methods require long reads or emulsion PCR. Here the match between barcodes and sequence is extremely good.**

**I think the method will be useful for the community, including our lab.**
**I have just very minor comments:**

We sincerely thank reviewer 2 (Olivier Tenaillon) for its enthusiasm about the impact of our paper and all their positive comments on our work! We hope that our responses will clarify certain details of our paper's methodology and make the manuscript more accessible. Below, we provide detailed responses to each point, and we have revised the manuscript accordingly to improve clarity and precision.

## Comment #2.1. Twice it is mentioned: "multiple reactions and transformations were performed for each fragment" to reach the good numbers... It would be good to give a few examples of what multiple typically means... 3, 6, 20?

Response:

The reviewer is right to say that our initial description was inaccurate. Indeed, cloning and transformation efficiency varies slightly between fragments of the same gene (sequence complexity, T/A stretch, GC%, etc.). To provide more concrete information, we now specify the average numbers used in the library construction:

- **Gibson assembly**: typically, 2 reactions (10 μL each), followed by ~5 transformations (3 μL of assembly reaction per transformation) using NEB competent cells.
- **Golden Gate assembly**: typically, 2 reactions (20 μL each), followed by ~10 transformations (2.5 μL of assembly reaction per transformation) using NEB competent cells.

Methods section, *started line 446*:

Methods section, *started line 463*:

This additional information should make the protocol more transparent and reproducible for the community.

## Comment #2.2. The association from barcode to sequence is in theory imposed in the design. Here, a careful step of sequencing is done to validate it. Could it be possible to know the amount of differences between the initial coupling and the one observed. In other words, what would be the error rate if this "association sequencing" was not done and we jsut trusted the oPools sequences.

Response:

We agree with the reviewer that the association between barcodes and mutations is, in principle, dictated by design. However, as our strategy relies on degenerate oPools (to reduce synthesis costs), each oPool construct contains nine random nucleotides (the maximum allowed for each commercially synthesized oPool):

•	Three for the NNK codons that encode the specific amino acid substitutions within the targeted coding region (fragment X).

•	Six random nucleotides distributed throughout the barcode region, between two fixed barcode segments that uniquely identify the targeted codon.

Given this design, sequencing after Gibson cloning is necessary to confirm the precise association of each barcode with a given mutation.

To clarify this, we have slightly modified the main text explaining the necessity of the first sequencing:

Legend of Figure 1-A, *started line 102*:

Main text, *started line 185*:

However, it is important to note that, without this initial sequencing, the two fixed barcode segments would theoretically allow reliable identification of the position of the mutated codon (without knowing the exact type of mutation).

To evaluate the accuracy of this approach, we analyzed the post-Gibson sequencing of all the libraries of *PDR1,* that we have now constructed to answer some reviewers' comments. This confirmed that the designed association between fixed barcode regions and fragment position was maintained in over 97.1% of cases. However, the association fixed barcode regions and exact codon position varied a lot and was maintained in between 47.6 and 83.4% of cases. We have added this analysis in the Supplemental Table H.

**Overall it is a nice experimental protocol, that will be valuable for the community. Some, may be cheaper; alternative may exist but they do not include the coupling to barcodes, and end up having very large fraction of wild type sequences, which in the end make them much less cost competitive.**

**Olivier Tenaillon, I always sign my reviews.**

# Reviewer #3 (Guillaume Cambray, identifies himself): The manuscript "Making deep mutational scanning accessible: a cost-efficient approach to construct barcoded libraries for genes of any length" by Jann et al. describe an ingenious method to generate high-coverage variants libraries from relatively small pool of synthetic oligonucleotides. The authors present a proof-of-concept application, screening variant of yeast's PDR1 protein for increased antifungal resistance.

**The method consists in using pools of degenerate oligonucleotides associating NNK random variations at each codon with partially random barcodes to generate trackable diversity through subsequent barcode-to-mutation mapping, and barcode frequency analysis upon functional screening. This enables substantial economy of scale in term of the number of oligonucleotides to synthetize. The authors further describe a two-step cloning strategy that enable targeting sequences that exceed the size of available oligonucleotide pools. Combined, these two aspects provide a convincing method to cost-efficiently generate large libraries of mutants. Although the authors focus on protein coding sequences, variations of the approach could be extensible to any kind of sequences of interest.**

Response:
 We sincerely thank Reviewer #3 (Dr. Guillaume Cambray) for the constructive evaluation of our manuscript and the many insightful comments provided. We are grateful for the positive assessment of our methodological approach and for the thoughtful suggestions that have helped us improve the clarity and overall quality of the work. The reviewer raised several important points regarding the scope of the method, the level of methodological detail, and areas where additional clarification or discussion could strengthen the manuscript. We have carefully addressed these points and revised the text accordingly. We believe these changes have significantly improved the manuscript. Below, we provide detailed point-by-point responses and indicate where revisions were made.

## Comment #3.1. The method is conceptually sound and its efficiency demonstrated experimentally for two small and separate regions of PDR1. In my opinion, the manuscript nevertheless fails to demonstrate the applicability of the method to "genes of any length", as claimed in the title and repeatedly in the text. In effect, the manuscript only provides data for one sub-library covering 75 nucleotides of a 3,204-nucleotide gene (and in a more limited manner on another sub-library of 54 nucleotides).

**While the core concept is appropriately demonstrated and it is implicitly stated that it can be easily scaled up, I do not think it does. I happen to be working with a similar strategy for constructing variants of a 6 kb genome—which yielded 45 sub-libraries—and I know for a fact that mixing these sub-libraries into a full library to perform pervasive screens leads to all sort of unforeseen issues. I therefore recommend that the manuscript demonstrate application to a sizable sequence length to substantiate one of the main claims of the paper. This might not need to be on the full extent of the pdr1 gene, but at least on a substantial length that exceed pure synthesis capacity (say 1kb).**

Response:

We thank Reviewer #3 for raising this important point regarding the scope of our method and the evidence supporting its applicability to genes of any length. We agree that demonstrating scalability beyond short regions will help support one of the main claims of our study.

To address this concern, we have now constructed comprehensive mutant libraries covering the entire *PDR1* coding sequence, composed of 43 individual fragments spanning 1067 amino acids. The results of this large-scale library construction are now included in the revised manuscript and supplementary materials. Specifically, Supplementary Table 8 summarizes key metrics for each fragment, including at each cloning step (Gibson and Golden Gate) the number of transformants obtained, the number of sequencing reads, the number of informative barcodes, the percentage of mutation coverage across different barcode thresholds (>0, >4, >9), and the Gini coefficient as a measure of variant distribution uniformity within each sub-library.

As for pooling different sub-libraries, this is something we have done in the past for a different type of construction, for instance when we were constructing libraries codon-by-codon and then pooling them into fragments from screening ([[3,4]](https://paperpile.com/c/Ryldux/hcY3q+uDId) for instance,). We have not seen any problem, at least at the scale we were working with. It may not be useful anyway to pool the whole library for screening since it will be highly diverse and may demand to work with large volumes of culture. One may choose to create a few small sub-libraries for some fragments while having fragments present in multiple pools to allow for normalization among pools.

Manuscript, *started line 553*:

Furthermore, we now provide a visual representation of these data in heatmaps illustrating mutation coverage and barcode diversity per position after Gibson cloning (Supplementary Figure 7) and after Golden Gate assembly (Supplementary Figure 8).

Supplementary Figure 7

**Supplementary Figure 7: Library quality control after Gibson assembly of the full-length *PDR1* sequence (43 fragments).** Heatmap displays the barcode diversity associated with each possible amino acid substitution at each codon position, with values clipped at 10 barcodes per mutation for clarity.

Supplementary Figure 8

**Supplementary Figure 8: Library quality control after Golden Gate assembly of the full-length *PDR1* sequence (43 fragments).** Heatmap displays the barcode diversity associated with each possible amino acid substitution at each codon position, with values clipped at 10 barcodes per mutation for clarity.

Together, these results demonstrate that our approach can be reliably scaled to the full length of a large gene and that it maintains high mutation coverage, barcode diversity, and a good uniformity across all sub-libraries.

## Comment #3.2. I understand and respect that the authors might want to valorize their perhaps more extensive work with several publications, with the present manuscript being more method-oriented. I seem to understand that the authors chose to focus on a well-known region where mutants are already known and want to keep extensive descriptions of screening results on other regions for another functionally-oriented paper. There might be ways to present these data without focusing on (or even disclosing) their biological meaning, just showing that many sub-libraries can be constructed, pooled sufficiently uniformly to be assayed together and quality functional data be obtained over large sequences.

Response:

We thank the reviewer for this comment and for recognizing the rationale behind focusing this manuscript on the methodological aspects of library construction. We agree that presenting the full breadth of functional screening data would go beyond the intended scope of the present study, which is specifically aimed at describing and validating the strategy for cost-efficient construction of barcoded mutant libraries.

As noted by the reviewer, the broader application of this approach to large-scale screening experiments will be presented in a separate, function-oriented manuscript. Our goal here was to provide the community with a reproducible framework for library construction and to highlight the advantages of incorporating internal replicates through barcode design, which we believe are of particular methodological importance. We also wanted to do this early as there is an expanding need for affordable approaches in the community. Waiting for a full paper oriented on the biology of *PDR1* would have taken much more time and delayed this publication.

Importantly, this strategy is not restricted to building complete gene libraries. It can also be applied in a flexible manner to construct sub-libraries targeting few dispersed hotspot regions within a large gene, which can then be pooled for functional screening. This provides an efficient way to investigate specific domains of interest while maintaining the advantages of low-cost construction and internal replication.

Therefore, we believe it is best to focus this short article on the methodology for effective mutant construction, while providing proof-of-concept libraries for part of the gene. This lays the foundation for future studies on large-scale functional screening.

Nevertheless, we have now completed the library construction to show that the approach applied to the fragments initially presented works well with all gene fragments. We believe that the rest of the application would be more standard as it is typical for certain types of libraries to be constructed piecewise and then pooled. For instance, in our recent paper [[3]](https://paperpile.com/c/Ryldux/uDId), the 200 codon library was constructed codon-by-codon and then pooled into sub-libraries.

**This being a method paper, I would also recommend to strengthen the precision of the materials and methods section.**

**I have listed below a number of additional comments that may help to improve the manuscript:**

**Figure 1:**

## Comment #3.3. Panel A: some aspect of the construction scheme could be depicted with more precision, e.g. including elements that are brought by oligonucleotides during PCR on the oligos. Some elements are also at odd with the description on the metod section (see details below)

Response:

We thank the reviewer for this helpful suggestion. We have slightly revised Figure 1A, particularly the section illustrating the oPool design, to provide clearer information on the flanking sequences and their respective roles, without overcomplicating the schematic. We also expanded the figure legend to include these details and now refer explicitly to the Supplementary Table 1, which lists all oPool sequences in detail.

Figure 1A:

Legend of Figure 1A, *started line 87*:

Importantly, we note that the PCR step performed on the oPool does not introduce any additional elements; all necessary components are already integrated into the oPool design. We believe that the revisions described above help clarify this point. Additional details regarding oPool composition and design are provided in Supplementary Table 1, which lists all oPools sequences in detail.

## Comment #3.4. Panel B: Reading the main text, it was particularly unclear to me where the data shown here came from. I seem to understand from the previous section that these are derived by cumulating sequencing sequencing data obtained separately on independent small-scale transformations. This should be stated clearly in the text. Points should be added to the graphs in addition to lines, since it is difficult to tell where the straight lines break.

Response:

We agree that the data shown in Figure 1B was not sufficiently clear. We have revised the Figure 1B legend and the main text to explicitly state that the presented data are based on the cumulative sequencing results from independent small-scale transformations. Furthermore, to make the visualization clearer, we have added data points on the graphs in addition to the existing lines.
We also clarified that results are presented as the number of transformants per base pair to enable meaningful comparisons across fragments of different lengths, making it easier for other researchers to apply these results to their own genes of interest.

Figure 1B:

Legend of Figure 1B, *started line 110*:

Main text, *started line 204*:

## Comment #3.5. The denomination "% barcode diversity" was somehow difficult to grasp. I seems to understand that this quantity represents the percentage of sequence associated with at least 4 and at least 9 informative barcodes? Maybe something like #barcode>4 and #barcode>9 could be clearer (this also apply to panel CD).

Response:

We thank the reviewer for this suggestion. Following this comment, we have revised the definition of “barcode diversity” throughout the manuscript to make it clearer and more intuitive, as recommended. Specifically, we now define barcode diversity as the percentage of mutations represented by #barcodes >4 or >9 ). To reflect this clarification, we have updated the terminology consistently across the text, figures, methods, and figure legends. For example:

- **Main text:** “Mutation coverage (i.e., percentage of mutations represented by at least one informative barcode); Barcode diversity (i.e., percentage of mutations represented by informative #barcodes >4 or >9 ), which provides internal replicates”
- **Figure 1B legend:** “barcode diversity (percentage of mutations represented by informative #barcodes >4 or >9 )”
- **Figure 1C–D legend:** “Covered mutations are mutations represented by #barcode >0, while barcode diversity represented by #barcode >4 or >9 per mutation demonstrates an increasing number of replicates for a same mutation.”
- **Methods section:** “Metrics included: (i) informative barcodes; (ii) mutation coverage; (iii) barcode diversity: #barcodes >4 per mutation; and (iv) barcode diversity: #barcodes >9 per mutation.”
- **Figures:** Labels were changed from “% barcode diversity for mutations represented by >4 barcodes” to “% mutations represented by #barcodes >4 ” and from “% barcode diversity for mutations represented by >9 barcodes” to “% mutations represented by #barcodes >9 .”

We hope these changes will improve the readability of the manuscript and will make the interpretation of barcode diversity more intuitive.

## Comment #3.6. l102: "Means and confidence intervals obtained for 100 random draws of transformation reactions": I am not sure I understand what this means exactly.

Response:

We thank the reviewer for pointing out the ambiguity in this sentence. It has now been revised to:

“Means and confidence intervals were obtained from 100 random draws of randomly picked transformants.”

## Comment #3.7. Panel CD: the thremomter should show discretized colors and the color scheme should make it easier to tell exactly how many barcodes are identified in each position. Data on the rightmost panel would be better shown as a barplot.

Response:

We have updated the heatmaps to use a discretized color scale, making it easier to distinguish the number of barcodes per mutation position.

Because the main goal of this figure is to demonstrate that most mutations are represented by >4 barcodes, the minimum threshold we determined necessary for robust correlation, we used a scale optimized to highlight barcode counts from 0 to 10.

Figure 1 - CD:

In addition, to improve transparency and address the reviewer’s comment, we have included supplementary figures (Supplementary Figures 1 and 2) in which the unlimited number of barcodes per mutation is displayed.

Supplementary Figure 1

**Supplementary Figure 1: Barcode diversity after Gibson assembly.** Heatmaps show the total number of unique barcodes for each possible amino acid substitution at each codon position in *PDR1* fragments F13 and F43. For each fragment, a total of 25,000 transformants were recovered and analyzed. Some amino acids, such as serine, leucine, and arginine, are more accessible with the NNK library (six codons each instead of four) and thus show higher diversity. Grey squares indicate wild-type (WT) amino acids.

Supplementary Figure 2

**Supplementary Figure 2: Barcode diversity after Golden Gate assembly.** Heatmaps show the total number of unique barcodes for each possible amino acid substitution at each codon position in *PDR1* fragments F13 and F43. For each fragment, a total of 100,000 transformants were recovered and analyzed. Some amino acids, such as serine, leucine, and arginine, are more accessible with the NNK library (six codons each instead of four) and thus show higher diversity. Grey squares indicate wild-type (WT) amino acids.

These two new figure were mentioned in the legend of Figure 1, *started line 124*:

Regarding the reviewer’s suggestion that the data in the rightmost panel would be better represented as a bar plot, we agree that a heatmap is not the most informative way to present this information. The corresponding data feature in the main text, where the F13 and F43 libraries are described.

Main text, *started line 545*:

## Comment #3.8. Figure 2 Panel B: I understand that a heatmap conveys more information per se, but comparing data from direct and barcode-based mutant identification would be better conveyed with a scatter plot of the one vs the other. Points could be additionally colored by the number of barcodes from which the values are estimated for the barcode-base identification. The heatmap for direct sequencing is not necessary and could either be removed or kept is space allows.

**More generally, an explanation of the few observed discrepancies between the two approaches could be discussed.**

Response:

We thank the reviewer for this helpful comment. We agree that a scatter plot is an effective way to compare direct variant identification and barcode-based estimates. We have therefore added a scatter plot comparing the two approaches with points colored according to the number of barcodes supporting each variant (Supplementary Figure 6). We retained the heatmap in the main figure as it is more comprehensive, but the scatter plot is now included as Supplementary Figure 6, showing a strong correlation (r = 0.97, all available barcodes).

Supplementary Figure 6

**Supplementary Figure 6: Correlation between the selection coefficients measured with direct sequencing of the mutated *PDR1* F13 coding region and DNA-barcode inference, using all usable barcodes.** The strong correlation (Pearson *r* = 0.97) indicates that barcode-based estimates accurately recapitulate the directly measured fitness effects. The number of barcodes per mutation does not affect the correlation.

We have also expanded the main text to briefly address and interpret the few discrepancies observed between the two approaches. Only a few discrepancies between barcode- and sequence-based estimates were observed (8 out of 525 mutations), most of which corresponded to variants located in the second half of fragment F13 (region not associated with resistance) and involved mutations with few barcodes (≤ 5) or neutral-to-sensitive fitness effects. These differences likely reflect stochastic noise in low-replicate measurements.

Main text, *started line 283*:

## Comment #3.9. The NNK strategy can yield one stop codon at each position. The heatmap from direct sequencing show occasional positive selection for these, while that from direct identification show mildly negative values. I would expect highly negative selection for these mutations and think that the observed data deserve some explanation: is it expected that truncated protein remain functional? Is it due to leakage from the endogenous gene that is normally dominated by the plasmid-borne variants? Is it indicative of a lack of measurement precision?

Response:

We appreciate this important observation and have clarified the underlying biological context. *PDR1* is not an essential gene, and several efflux pumps regulated by Pdr1 can also be activated by other transcription factors. Therefore, loss-of-function mutations in *PDR1* do not necessarily lead to high sensitivity to posaconazole, and we expected them to be neutral or slightly deleterious.

Main text, *started line 230*:

Moreover, in the original direct sequencing data, three positions (306, 309, 323) showed slightly positive values (median across positions: 0.05-0.1), likely due to measurement noise. The detection threshold was higher in the barcode-based analysis, where replicate averaging across multiple barcodes improves measurement precision. However, after implementing other reviewer recommendations, we reanalyzed all data. Now, all stop codon variants in the PDR1 F13 sequence and in the barcode analysis display negative selection coefficients, as expected (Revised Figure 2 - B). To better illustrate this point, the following Figure D shows with a violin plot the distribution of selection coefficients for all STOP codons under posaconazole treatment for both analyses (*PDR1* F13 sequence and barcode).

*Figure D*:

**Figure D: Violin plot of the distribution of selection coefficients for all STOP codons under posaconazole treatment for both analyses (*PDR1* F13 sequence and barcode).**

Figure 2 - B:

## Comment #3.10. Panel C: l204-205: "Means and confidence intervals were calculated from 100 random subsamplings per variant." I do not see error bars here. Also, it is unclear how these data are generated: are variants with more or less than N barcodes left out from the correlation? If so, the correlation obtained by using all usable data should be communicated.

Response:

We thank the reviewer for this comment. We confirm that error bars are indeed present in the figure. If they appear invisible, it is because the associated variation is extremely small and therefore difficult to visualize. To address this, we revised the figure using boxplots to better illustrate the narrow distribution of correlation coefficients obtained across the 100 subsamplings.

Figure 2 - C

**Figure 2 - C) Distribution of correlation between the selection coefficients measured with direct sequencing of the mutated F13 coding region and DNA-barcode inference**, as a function of the number of barcodes per variant considered. Each boxplot summarizes 100 random subsamplings per variant, performed using a standardized resampling procedure to normalize barcode counts across variants.

We also confirm that all variants were included in the correlation analyses. If a variant had fewer barcodes than the target number, some barcodes were resampled multiple times to reach the desired number of barcodes. If a variant had more barcodes than required, a random subset was selected for the analysis. To further clarify this analysis step, we have added a dedicated paragraph in the *Methods section* of the revised manuscript describing the barcode subsampling strategy in detail.

Method section, *started line 744*:

## Comment #3.11. l148: "The performance of a DMS plasmid library depends on three key parameters": I do agree with all these parameters but would argue that a fourth parameter quantifying representation bias between variants is also key. Such bias can come from synthesis, construction, subsequent growth, etc… Significant distribution biases hinder cost-effectivess as much sequencing throughput is wasted on the most abundant to get data for the least abundant variants. A metric should be chosen to quantify this.

Response:

We agree with the reviewer that representation bias is an important parameter. As described in our response to to comment #1.2), we now include the Gini coefficient and uniformity index as quantitative metrics to assess distribution bias across variants.

## Comment #3.12. l218-219: "estimates from the barcodes or from the F13 coding region were strongly correlated (r = 0.95, 10 barcodes)": it would be better to highlight the correlation when all available barcodes are used.

Response:

We appreciate this suggestion. When all available barcodes are used, the correlation between barcode and direct *PDR1* F13 sequencing increases to r = 0.97. We have updated the text accordingly and added a new Supplementary Figure 6 illustrating this correlation (Pearson) for the selection coefficients under posaconazole treatment.

Main text, *started line 286*:

Supplementary Figure 6:

**Supplementary Figure 6: Correlation between the selection coefficients measured with direct sequencing of the mutated *PDR1* F13 coding region and DNA-barcode inference, using all usable barcodes.** The strong correlation (Pearson *r* = 0.97) indicates that barcode-based estimates accurately recapitulate the directly measured fitness effects. The number of barcodes per mutation does not affect the correlation.

## Comment #3.13. l342-343: "Since oPools are synthesized in the 5' to 3' direction, this initial step minimizes the incorporation of truncated oligos (linked to oPool incomplete synthesis) into the final product": this is factually wrong, the phosphoramidite chemistry used for all chemical oligonucleotide synthesis actually proceeds in the 3' to 5' direction. This first PCR and associated purification seems rather useless.

Response:

We thank the reviewer for pointing out this important correction. The reviewer is correct that oligonucleotide synthesis proceeds in the 3′ to 5′ direction, not 5′ to 3′ as originally written. We have corrected this in the revised manuscript. Our intention was not to describe the chemical directionality of synthesis, but rather to clarify that oPools contain a fraction of truncated oligos due to incomplete synthesis and find a solution to reduce their amount.

Method section, *started line 425*:

## Comment #3.14. l348-350: "This approach improved the overall quality of the oPools, ensuring sufficient insert quantity and diversity for subsequent steps of DMS library construction.": I don't think this claim is substantiated by data, please edit to something along the line of "This approach is intended to improve..."

Response:

We have edited the sentence as suggested:

“This approach is intended to improve the overall quality of the oPools, ensuring sufficient insert quantity and diversity for subsequent steps of DMS library construction.”

## Comment #3.15. l357-359: "The resulting PCR product was incubated for 1h30 at 37 °C with 10 U of DpnI enzyme to remove parental plasmid DNA and then, purified using magnetic beads.": In this description the linear PCR product is directly used for Gibson assembly. This is not what is suggested in Figure 1A, where it appear as a circular intermediate obtained from transformation (which would have been better to avoid PCR-related mutation in the backbone, including part of YFV). Likewise for the second cloning step, the missing fragment would have better been cloned along with flanking BsaI and sequence verified before used in golden-gate. I suggest the author add such recommendations to the method section as potential improvements.

Response:

We thank the reviewer for the detailed suggestions. We would like to clarify that Figure 1A indeed illustrates PCR amplification, as shown by the primer arrows and the explicit mention of “PCR.” Moreover, the DpnI treatment step is included only to remove the parental plasmid template. To further clarify this point, we have modified the *Methods section* as follows:

Methods section, *started line 440*:

Regarding the concern about PCR-induced mutations, all PCR reactions in this study were performed using a high-fidelity KAPA HiFi HotStart polymerase, as clarified now in the Methods section, *started line 329*:

In addition, we performed quality control sequencing on six plasmids from six independent fragments after Golden Gate assembly. This sequencing confirmed that no unintended mutations were present across the *PDR1* promoter, coding sequence, or terminator regions. These validation data have been added in the *Methods section*.

Method section, *started line 574*:

Regarding the comment about the potential use of restriction sites to avoid PCR amplification of the backbone. However, one of the central goals of this work is to reduce cost and simplify the cloning workflow. Introducing restriction sites for each fragment would require designing and constructing 43 different backbones, which would significantly increase experimental complexity, time, and cost, and potentially introduce new sources of error (e.g., undesired restriction sites within the gene or plasmid).

Regarding the second part of the comment, we fully agree that sequencing each PCR product prior to Golden Gate assembly would provide additional quality control. However, given that the missing fragments ranged from 420 bp to 3500 bp, systematically sequencing all products at this stage would have been prohibitively expensive and would not have aligned with the cost-efficiency objectives of this study.

For these reasons, we opted for a strategy that balances accuracy, efficiency, and cost-effectiveness. However, as suggested, we have now included a few sentences in the Methods section noting that pre-sequencing of all PCR products could be considered as an optional additional quality-control step if desired (see text in method section above).

## Comment #3.16. l363-365: "Following transformations, 5 mL of 2YT medium was added directly onto each agar plate": this is too imprecise for a methodological paper. What transformation procedure is used (chemical, electroporation)? As I understand 2YT was added to the cell after the transformations. Were cell grown after that before plating. At what point are transformant counted?

Response:

We thank the reviewer for this comment and the opportunity to clarify this point. The highlighted sentence was simply meant to describe how the transformants were collected. Specifically, 5 mL of 2YT medium was added directly onto each agar, colonies were scraped with a glass rake and collected in a tube. All colonies coming from a single library fragment were pooled together. These pooled transformants were subsequently used to prepare glycerol stocks and cell pellets for miniprep plasmid extraction, thereby recovering the complete plasmid library corresponding to each library fragment.

Importantly, no additional bacterial growth step was performed after transformation.

These informations are mentioned the Methods section, *started line 450*:

Additionally, we have now included a short description of the transformation procedure used (see below).

Method section, *started line 334*:

## Comment #3.17. l380-381: "To achieve this, multiple parallel Golden Gate reactions and transformations were performed for each fragment." This is surprising to me, a transformation efficiency of 10^5 is fairly easy to attain, especially with commercial competent cells...

Response:

We thank the reviewer for this observation. In our experiments, the transformation efficiency following Golden Gate assembly was approximately 10⁴ transformants per reaction when using commercial competent cells. To achieve the desired library complexity, we therefore performed around 10 parallel transformations per fragment. These details have now been added to the Methods section for clarity.

We have added these details to the methods section, *started line 446*:

Methods section, *started line 461*::

## Comment #3.18. 389-… Plasmid library quality control by sequencing: Description of the the sequencing libraries preparation comes after description of sequencing read processing. It would be clearer to have a dedicated section for the various flavors of sequencing library preparation.

**More broadly, amplicon sequencing is notoriously biased, with random molecules sometime getting way more amplified than other, which is a big problem for downstream quantitative analyses. A now common approach to mitigate this is to incorporate Unique Molecular Identifier during PCR. The authors have not done this here, but could discuss this as a weakness and suggested it as an improvement.**

Response:

We thank the reviewer for this valuable suggestion, which improves the clarity and rigor of the manuscript. We agree that the description of sequencing library preparation should logically precede the description of sequencing read processing. We have therefore restructured this section accordingly in the revised manuscript, so that the library preparation protocols are now described in a dedicated subsection before the bioinformatic processing steps.

Regarding the use of Unique Molecular Identifiers (UMIs) to minimize amplification bias during amplicon sequencing, we appreciate this important point. As the reviewer correctly notes, we did not use UMIs for the sequencing of barcode libraries generated after Golden Gate assemblies, primarily due to technical constraints imposed by our design: the PBS_i5 and PBS_i7 priming sites directly flank the barcode region and are required for rapid and efficient library preparation. The reproducibility of our results suggest that overamplification of specific fragments in PCR reactions was not an issue.

However, we agree that incorporating UMIs could be a valuable improvement, particularly during quality-control sequencing of the mutated coding fragment after Gibson cloning, where amplification bias may be highly relevant. We have added a discussion of this point in the revised manuscript and highlighted the possibility of using UMIs as an optional refinement for future implementations of this approach.

Method section, *started line 475*:

## Comment #3.19. l446-....: "Variant library construction": most of this description is not matched by any main text. A few main text sentences describing that PDR1 is essential, has been placed under a dox-repressible promoter to enable turning its expression down and conditional complementation by a plasmid library would be welcome.

Response:
We thank the reviewer for this helpful suggestion. As clarified in the revised manuscript, *PDR1* is not an essential gene: cells remain viable in its absence.

Main text, *started line 230*:

To better contextualize the “Variant library construction” section, we have now added sentences in the main text explaining the experimental design. Specifically, we highlight that the goal of the variant library was to functionally characterize the effects of *PDR1* mutations in a controlled genetic background. To achieve this, the native *PDR1* promoter was replaced with a doxycycline-repressible promoter, allowing us to control the genomic expression of *PDR1* and perform conditional complementation with plasmid-encoded variant libraries.

This design choice was also motivated by practical considerations: previous attempts to generate a complete genomic deletion of *PDR1* resulted in very low transformation efficiency. Using a conditional promoter bypasses this limitation while still making it possible to repress the expression of genomic *PDR1* expression. These clarifications have been incorporated into the main text and the method section of the revised manuscript.

Main text, *started line 271*:

Method section, *started line 583*:

## Comment #3.20. l535-536: "an initial PCR was carried out to amplify only the PDR1 from the plasmid": I understand that this is necessary for the direct sequencing of F13 variants, and it makes sense to use the same material for barcode library prep in the framework of comparing data derived from barcode sequencing and direct variant region sequencing. However, higher quality data on barcodes could be derived by directly targeting PBS_i5 and PBS_i7 priming site...

Response:

We agree that directly amplifying the barcode region using the PBS_i5 and PBS_i7 priming sites would represent a more straightforward and potentially higher-quality approach for barcode sequencing.

In the present study, however, we performed an initial PCR on the plasmid *PDR1* region to enable a direct comparison between barcode-derived data and variant-derived data using the same sequencing material. This ensured that any differences observed between the two approaches were not due to differences in sample preparation. We now explicitly mention in the revised manuscript that, in a standard DMS experiment focused only on barcode tracking, direct amplification of the barcode region using PBS_i5 and PBS_i7 would be the most efficient option.

Method section:

## Comment #3.21. l563-...: "Selection coefficient and characterization of the variants". The authors have already established a pipeline to identify barcodes. As the very same pipeline can presumably be used to count barcodes, what exactly is the added value of using gyoza?

Response:

A first custom script was used to obtain barcode-variant associations. This step occurs during library preparation. gyoza was specifically designed to analyze DMS data and its latest version (revised at the same time as this manuscript) is now capable of analyzing unscreened and/or screened libraries, as long as the barcode-variant associations are provided. While the initial custom script only associates barcodes and variants, gyoza is a much more complete pipeline, providing many diagnostics graphs on top of converting the read counts into functional impact scores.

## Comment #3.22. l577: "normalization by the number of mitotic generations": this needs to be explained.

Response:

The text was modified to: “normalization by the number of mitotic generations, to account for different growth rates across cultures and make sure functional impact scores reflect the per-generation selective advantage, making them selection coefficients”.

## Comment #3.23. l578: "subtraction of the median log2 fold-change of silent variant": likewise, this require additional explanation. What is the fold-change distribution of silent variant, where is the actual WT in this distribution? How does it compare with the distribution of variants?

Response:

The average (median) log2 fold-change in allele frequencies of all mutants encoding the wild-type protein sequence (silent mutants), except for the true WT (wild-type nucleotide sequence), is used to normalize the values. We discuss this normalization in the gyoza paper (PMID 40976584). Briefly, the true WT is omitted because it is often overabundant in oPool designs and because using all silent mutants as reference provides more data points. Where the true WT lies compared to the distribution of silent mutants provides little information since this data point is simply not considered in downstream analyses. The spread of values for silent mutants (compared to single mutants) is illustrated in one of the diagnostic plots generated by gyoza, which uses 1 standard deviation (above and below the mean) as error band (Figure E).

Figure E:

**Figure E. Posaconazole selection of *PDR1* F13 mutants through time.** The distribution of selection coefficients (s) at time points T1 and T2 for mutants with a single amino acid change compared to the wild-type protein sequence of F13 is shown as a blue line (mean) and band (standard deviation). Red lines indicate the values obtained for the WT (average of silent mutants, excluding the true WT). Dashes and markers indicate replicates.

## Comment #3.24. l581-583: "Variants were assigned confidence scores based on their initial abundance at TP0 across replicates. A minimum threshold of five reads per mutation was used to define high-confidence variants": Does this mean that all replicates must have at least 5 reads? As far as I can tell, there has been no mention of experimental replicates until this point: what are they?

**As much as the number of initial reads is important, this seems a little light to qualify as a confidence score... I guess the spread of values around barcode replicates and synonymous amino-acids could be used for that purpose, but neither is mentioned, despite means being considered (l584).**

Response:

This “confidence score” indeed reflects the initial read count across replicates **when there are multiple replicates**. Unfortunately, a lack of coverage meant we needed to select a single replicate for the T0 sample. Since fold-changes are obtained by comparing matching replicates, we could not include the other replicates from output time points. The text has been modified accordingly. The reason why this is called a “confidence score” by gyoza is because the pipeline is meant to be easy to use, with an equally easy-to-interpret output. Since the pipeline handles multiple types of designs, this is also a way to quickly show the impact of initial read counts of estimates of mutational effects, regardless of the experimental design.

## Comment #3.25. Finally, a potential weakness of the strategy could be discussed. In the construction scheme, each codon position is associated with a defined barcode core of 24 nts, while 6 barcode positions are degenerate. One can't really rule out a potential functional effect of the core barcode, which would then systematically impact all variants at that position. Are there ways to diagnose or mitigate such effects?

Response:

We thank the reviewer for highlighting this potential limitation.

However, the barcode cassette is physically and transcriptionally isolated from the coding sequence. After the two-step cloning, the barcode is downstream of the *PDR1* terminator (around 345 pb) and within the PBS_i5/PBS_i7 flanking region (i.e., outside the transcribed ORF and 3′ UTR used for expression). As such, the barcode should neither be transcribed nor translated. Therefore, it is not expected to influence protein expression, stability, or function.

We nonetheless agree that a potential positional effect of the fixed barcode core, shared by all barcodes associated with a given codon, is worth considering. There is some swap between the codon and the barcode in the libraries such that the same codons are present with more than one fixed, position-barcode pattern. This allowed us to directly evaluate whether the fixed barcode regions could have a functional impact on the measured phenotype. To do so, we calculated the pairwise correlation of the median selection coefficients between two core barcodes associated with the same mutation. We observed a median Pearson correlation of 0.73 (interquartile range: 0.67 - 0.77). This value is comparable to the pairwise correlations observed among barcodes corresponding to the same variant (see response to comment #1.7), indicating that the fixed barcode regions do not introduce a systematic bias in our measurements.

# Reviewer #4: Systematic mutational scanning is a powerful technique that enables researchers to assess the functional impact of all possible point mutations in a gene within a single experiment, providing a comprehensive view of the relationship between protein sequence and function. However, generating libraries for these experiments is often prohibitively expensive and labor-intensive, with costs increasing proportionally to gene size.

**The manuscript by Jann et al. introduces a cost-effective and accessible method construction of a barcoded library that addresses these challenges. Overall, the manuscript is clearly written and well-presented, and the authors' elegant approach to library design will be valuable for future DMS studies. I have just a few comments and questions that arose while reading the manuscript. Overall, I recommend this manuscript for publication in PLOS Biology.**

Response:

We sincerely thank Reviewer #4 for its evaluation of our work and for the encouraging comments regarding its significance and clarity. We greatly appreciate the constructive suggestions, which have helped us to improve the precision and clarity of the manuscript. Below, we provide point-by-point responses and indicate where corresponding changes have been made in the revised version.

## Comment #4.1. It may be helpful to include a table summarizing coverage statistics for each of the two library fragments constructed in this study. This could include the number of transformants for the two cloning steps, sequencing reads, informative barcodes, mutation coverage, barcode diversity, and other relevant metrics.

Response:

We thank the reviewer for this suggestion. We agree that a summary table of coverage metrics would make the dataset clearer and more accessible. As requested, we have now included Supplementary Table 8, which summarizes the key statistics for each of the library fragments generated in this study. The table reports for each cloning step (Gibson and Golden Gate) the number of transformants, the number of sequencing reads requested and obtained, the number of informative barcodes, the percentage of mutation coverage for informative barcodes >0, >4 and >9, the Gini coefficient (uniformity), etc.

Additionally, because we ultimately constructed the full mutant library for the entire *PDR1* gene (43 fragments, 1067 amino acids total), we now include the data sets for each of these fragments in the same Supplementary Table 8. Summary of coverage statistics for the two cloning steps for all libraries of the complete *PDR1* gene.

Mentioned in the Method section, *started line 553*:

## Comment #4.2. While the oPool-based method is clearly more affordable than purchasing a commercial library, it appears potentially labor-intensive—particularly when synthesizing a large gene such as PDR1 in 43 separate fragments. Could the authors provide an estimate of how many days it would take to clone the entire PDR1 library using this strategy?

Response:

We appreciate this question and agree that providing an estimate of the overall experimental timeline would be useful to the reader. Below, we outline a typical timeline based on our experience with constructing the entire 43-fragment *PDR1* library:

- oPool and backbone amplification: ~1–2 weeks
- Gibson assembly: ~1 month
- Amplification of missing fragments: ~1 week
- Golden Gate assembly : ~2 months
- Library preparation for each sequencing (~1 week)
- Sequencing turnaround time (~1–2 months, depending on sequencing platform availability).

While this two-step strategy is more time-consuming than standard approach, it is important to note that cloning step is still required even with commercial library synthesis approaches. In such cases (standard approaches), the overall experimental timeline would typically be ~2 months, compared to ~4 months with our approach, but with a substantial reduction in costs and the possibility of building barcoded mutant libraries from any region of a gene, regardless of its size.

It is also worth mentioning that the total time required for library construction can vary depending on several factors, such as the complexity and sequence composition of the target gene, the total number of libraries to be constructed (one library per fragment of ~ 100pb), and how many people are involved in the experimental work. For information, this estimated timeline is based on the fact that most of the library construction was carried out by a single person.

All of this additional information has now been incorporated into a "Supplementary Table 9 - Estimate experimental timeline to construct the complete plasmid mutant *PDR1* library" to better guide readers who may wish to apply this approach in their own work.

Mentioned in the Method section, *started line 553*:

## Comment #4.3. In Supplementary Figure 1A, the cost of cloning a commercially synthesized library is estimated at approximately $60 per amino acid position. I was curious how this number was calculated? Based on a rough back of the envelope estimate—assuming ~100,000 transformants per Gibson assembly reaction and thus approximately 10 reactions to cover a 300-aa barcoded library—the total cost in my mind would only be a few hundred dollars in total. Could the authors clarify the basis of the $60/aa cost?

Response:

We thank the reviewer for pointing out that the basis for this estimate was unclear. The ~$60 per amino acid shown in the previous Supplementary Figure 1A (which was removed following a reviewer’s comment) did not represent an internally calculated cost. Rather, it reflects the price quoted by commercial providers (e.g., Twist Bioscience) for cloning a synthesized variant library directly into a vector, based on quotations we received for similar projects.

**References:**

1.	[Mateyko N, de Boer CG. Culture wars: Empirically determining the best approach for Plasmid library amplification. ACS Synth Biol. 2024;13: 2328–2334. doi:](http://paperpile.com/b/Ryldux/LxcX)[10.1021/acssynbio.4c00377](http://dx.doi.org/10.1021/acssynbio.4c00377)

2.	[Mighell TL, Toledano I, Lehner B. SUNi mutagenesis: Scalable and uniform nicking for efficient generation of variant libraries. PLoS One. 2023;18: e0288158. doi:](http://paperpile.com/b/Ryldux/mJgg)[10.1371/journal.pone.0288158](http://dx.doi.org/10.1371/journal.pone.0288158)

3.	[Bédard C, Gagnon-Arsenault I, Boisvert J, Plante S, Dubé AK, Pageau A, et al. Most azole resistance mutations in the Candida albicans drug target confer cross-resistance without intrinsic fitness cost. Nat Microbiol. 2024;9: 3025–3040. doi:](http://paperpile.com/b/Ryldux/uDId)[10.1038/s41564-024-01819-2](http://dx.doi.org/10.1038/s41564-024-01819-2)

4.	[Després PC, Cisneros AF, Alexander EMM, Sonigara R, Gagné-Thivierge C, Dubé AK, et al. Asymmetrical dose responses shape the evolutionary trade-off between antifungal resistance and nutrient use. Nat Ecol Evol. 2022;6: 1501–1515. doi:](http://paperpile.com/b/Ryldux/hcY3q)[10.1038/s41559-022-01846-4](http://dx.doi.org/10.1038/s41559-022-01846-4)
