# Supplementary material for: A cost-effective and scalable barcoded library construction method for deep mutational scanning studies
Source: PLoS Biol. 2026 Feb 11;24(2):e3003645. doi: 10.1371/journal.pbio.3003645 (PMC12923136; doi:10.1371/journal.pbio.3003645)
Supplement: Attachment — Submitted filename: Response_Round#2_Jannetal_PLOS_Biology.docx [file pbio.3003645.s037.docx]

**Peer Review Information - Round#2**

**Journal:** PLOS Biology as a *Methods and Resources Article*

**Previous Manuscript Title:** Making deep mutational scanning accessible: a cost-efficient approach to construct barcoded libraries for genes of any length

**Revised Manuscript Title:** A cost-effective and scalable barcoded library construction method for deep mutational scanning studies

**Corresponding author name:** Christian Landry

**Authors responses:**

**Comments From PLOS Biology editors:**

## **Comment #1.1. We routinely suggest changes to titles to ensure maximum accessibility for a broad, non-specialist readership. In this case, we would suggest the following edit to the title. Please ensure you change both the manuscript file and the online submission system, as they need to match for final acceptance:**

**“A cost-effective and scalable barcoded library construction method for deep mutational scanning studies”**

Response:

We agree with this comment and have now changed the title as follows: “A cost-effective and scalable barcoded library construction method for deep mutational scanning studies”

## **Comment #1.2. We note that the Abstract is currently 71 words and we would be grateful if it could be expanded at this stage to around 150 words to fully contextualize and summarize the methodological developments offered by the study.**

Response:

As requested, we have expanded the Abstract to 150 words to better summarize our cloning strategy, its scalability, and the experimental validation presented in this study:

Abstract:

“Recent developments in DNA synthesis and sequencing allowed the construction of comprehensive gene variant libraries and their functional analysis. Achieving high-replication and thorough mutation characterization remains technically and financially challenging for long genes. Here, we developed an efficient, affordable and scalable library construction approach that relies on low-cost DNA synthesis and standard cloning technologies, which will increase accessibility to mutational studies and help advance the field of protein science. Each degenerate codon variant is physically associated with multiple DNA barcodes during synthesis, which overcomes the need for long-read sequencing for linking variants to barcodes. We demonstrate the scalability of our approach by constructing a complete library for *PDR1*, a 3.2 kb multidrug resistance gene encoding a pleiotropic transcription factor in the yeast *Saccharomyces cerevisiae*. We demonstrate a near perfect correspondence in the measurement of amino acid variants impact when assessed by barcode sequencing and direct sequencing of the mutated coding sequence.”

## **Comment #1.3. You may be aware of the PLOS Data Policy, which requires that all data be made available without restriction: http://journals.plos.org/plosbiology/s/data-availability. For more information, please also see this editorial: http://dx.doi.org/10.1371/journal.pbio.1001797**

**Note that we do not require all raw data. Rather, we ask that all individual quantitative observations that underlie the data summarized in the figures and results of your paper be made available in one of the following forms:**

**-Supplementary files (e.g., excel). Please ensure that all data files are uploaded as 'Supporting Information' and are invariably referred to (in the manuscript, figure legends, and the Description field when uploading your files) using the following format verbatim: S1 Data, S2 Data, etc. Multiple panels of a single or even several figures can be included as multiple sheets in one excel file that is saved using exactly the following convention: S1_Data.xlsx (using an underscore).**

**-Deposition in a publicly available repository. Please also provide the accession code or a reviewer link so that we may view your data before publication.**

**Regardless of the method selected, please ensure that you provide the individual numerical values that underlie the summary data displayed in the following figure panels as they are essential for readers to assess your analysis and to reproduce it:**

**Figure 1B-D, 2B-C, S1, S2, S3, S4, S5B, S6, S9**

**NOTE: the numerical data provided should include all replicates AND the way in which the plotted mean and errors were derived (it should not present only the mean/average values).**

Response:

To follow PLOS Data Policy requirements, all individual quantitative data underlying the figures have now been made available either as Supporting Information files (S1_Data.xlsx, S2_Data.xlsx, etc.) and are clearly referenced in the manuscript and figure legends.

## **Comment #1.4. I appreciate that the data requested above may be contained within your Github deposition (https://github.com/Landrylab/Jann_et_al_2025). If so, I would be grateful if you could clearly label where the underlying data can be found in the deposition folders.**

Response:

As requested, we have now reorganized the Github deposition by Figure, in order to make it easier for readers to locate the underlying data in the deposition folders.

**Comment #1.5. Please note that we cannot accept sole deposition of code in GitHub, as this could be changed after publication. However, you can archive this version of your publicly available GitHub code to Zenodo. Once you do this, it will generate a DOI number, which you will need to provide in the Data Accessibility Statement (you are welcome to also provide the GitHub access information). See the process for doing this here: https://docs.github.com/en/repositories/archiving-a-github-repository/referencing-and-citing-content**

Response:

We agree. We have archived the current version of our publicly available GitHub repository on Zenodo, which generated the following DOI: 10.5281/zenodo.18342596. This DOI has now been included in the Data Accessibility Statement, together with the GitHub repository link.

## **Comment #1.6. Please also ensure that each of the relevant figure legends in your manuscript include information on *WHERE THE UNDERLYING DATA CAN BE FOUND*, and ensure your supplemental data file/s has a legend.**

Response:

We have updated all relevant figure legends to indicate where the underlying data can be found. In addition, when necessary, legends have been added to Supporting Information data files to describe their contents.

## **Comment #1.7. Please ensure that your Data Statement in the submission system accurately describes where your data can be found and is in final format, as it will be published as written there.**

Response:

The Data Statement in the submission system has been revised to describe the final locations of all data and code.

Data Statement in the submission system:

‘All data are available in the main text or the supplementary materials. The numerical data underlying all figures are provided in the files S1_Data.xlsx (corresponding to the Figure 1B), S2_Data.xlsx (corresponding to the Figure 1D, Supplementary Figures 2, 4, 6, 8 and 9), S3_Data.xlsx (corresponding to the Figure 1C, Supplementary Figures 1, 3, 5, 7 and 9), S4_Data.xlsx (corresponding to the Supplementary Figures 10 and 11), S5_Data.xlsx (corresponding to the Figure 2B and Supplementary Figure 13), S6_Data.xlsx (corresponding to the Figure 2B and Supplementary Figure 13), S7_Data.xlsx (corresponding to the Figure 2C), S8_Data.xlsx (corresponding to the Supplementary Figure 12B) and S9_Data.xlsx (corresponding to the Supplementary Figure 14). Sequencing data are available in the NCBI Sequence Read Archive (SRA) under BioProject PRJNA1269095. All code and processed data to reproduce the analyses, results and figures are available at <https://github.com/Landrylab/Jann_et_al_2025> and archived on Zenodo (DOI: 10.5281/zenodo.18342596).’

## **Comment #1.8. Please note that per journal policy, the species studied should be clearly stated in the abstract of your manuscript.**

Response:

We have revised the Abstract to clearly state the species studied (*Saccharomyces cerevisiae*), in accordance with journal policy.

**Comment from Reviewer #1:**

## **Comment #2.1. The authors have addressed most of my comments; they have added new experimental data scanning the entire gene. One very small note - in Figure 2B the PDR1 name is clipped by a white opaque box in the updated figure; this can be handled in the proof.**

Response:

We thank the reviewer for this comment. We have now revised Figure 2 so that in 2B the name *PDR1* is not obscured by a white box.

Revised Figure 2B:

**Comment from Reviewer #3 (Guillaume Cambray, identifies himself): This revised version of the manuscript "Making deep mutational scanning accessible: a cost-efficient approach to construct barcoded libraries for genes of any length" by Jessica Jann et al. largely addresses most of my previous questions and concerns, as well as those raised by the other reviewers.**

## **Comment #3.1. A major comment—also raised by another reviewer—concerned the manuscript's emphasis on the gene-scale applicability of the approach, while only two short regions were presented. The authors have now provided library construction data for all 43 regions of the gene (Sup. Figs. 7 and 8), which addresses this comment only in part.**

**The innovative aspect of this work lies in coupling NNK mutagenesis of a relatively short region (i.e., one that can be sequenced using widely available and cost-efficient short-read sequencing such as PE150) with a partially randomized barcode. As the authors point out, different barcodes attached to the same mutation can be viewed as internal replicates that enhance measurement accuracy and statistical robustness. Nevertheless, direct sequencing of the mutated region remains the gold standard, as used by the authors in Figure 2BC and Sup. Figs. 6 and 9.**

Response:

We thank the reviewer for this comment. We agree that the gold standard would always be to assay the mutation frequency directly. As discussed in the manuscript, this becomes infeasible for long genes. This is the reason why we developed a new approach.

**A major advantage of the barcode strategy is that multiple sub-libraries can be pooled and assayed simultaneously rather than separately, effectively performing one large experiment instead of 43 independent ones. Beyond the obvious cost efficiency, this pooling also improves data consistency and reduces the need for normalization. In my view, a complete validation of the proposed approach should demonstrate that a pool of sub-libraries can indeed be successfully screened. Without pooling, barcoding brings little added value: a single cloning of an NNK-mutagenized oPool, followed by sequencing of the mutated region—as performed here and in other studies (Tile-Seq)—would be sufficient.**

**In their response, the authors suggest that because they have successfully pooled sub-libraries previously, there is no need to demonstrate the effectiveness of pooling here. By that logic, however, there would have been no need to perform a screening experiment on a single barcoded sub-library, since barcoding itself is also a previously established strategy.**

Response:

Pooling and barcoding had indeed been used before and we decided here not to demonstrate their feasibility. Since the DNA synthesis strategy we used had not been used before, and the double-cloning steps were different, it was mandatory for us to demonstrate that the barcode-variant association was maintained throughout the construction process. We also needed to assess how reproducible the data would be from one barcode to another when compared to direct sequencing of the coding sequence. Since pooling various libraries does not involve DNA synthesis, PCR reactions, and cloning, it is much less prone to error than the steps that involve our library construction and that we chose to validate here.

**I emphasize this point because, in a very similar experimental context, pooling has unexpectedly caused issues in our own hands—even though we had previously pooled complex libraries successfully. This might be specific to our system, but it would be unfortunate if this manuscript did not address pooling experimentally to fully substantiate the method. I am not asking for a full gene-scale pool, but rather a small number of pooled regions (preferably contiguous, to highlight continuity in measurements across regions). This seems feasible considering all sub-libraries have already been constructed. Again, the goal is not an extended functional analysis—which could be published separately—but a demonstration that substantiates the method's core claim.**

Response:

We will follow the editorial decision here and not perform these additional experiments. We are currently planning on using the libraries to perform large-scale screening, in which we will show these results.

**Aside from this major point, I have a few remaining comments and suggestions, detailed below:**

## **Comment #3.2. * Lines 113-114:**

**"Means and confidence intervals obtained for 100 draws of randomly picked transformants."**

**Despite the revision, this statement remains unclear. To my understanding, a transformant refers to a single clone. The description in the Methods section is clearer. I suggest rephrasing along the lines of: "100 ordered arrangements of independent transformation experiments with 5,000 and 7,500 clones, respectively, were randomly drawn to construct this graph."**

Response:

As requested and using reviewer#3’s suggestion, we have now modified the sentence “Means and confidence intervals obtained for 100 draws of randomly picked transformants**.**” to “Means and confidence intervals were obtained from 100 random subsamplings of independent transformation experiments, each consisting of 5,000 (F13) or 7,500 (F43) transformants, cumulatively combined.”.

## **Comment #3.3. * Figure 1B:**

**Unless I am missing something, I do not understand what the statistics would indicate when the number of transformants per base pair is 0. I believe there may be an error in the x-axis labeling.**

Response:

We thank the reviewer for pointing this out. There was indeed an error in the code used to generate Figure 1B, which resulted in incorrect x-axis labeling. We have corrected the labeling, verified the normalization using the correct fragment lengths for both F13 and F43, and replaced Figure 1B with the corrected version in the revised manuscript.

## **Comment #3.4. * Lines 198-200:**

**"~350 transformants per base pair are needed to optimize sufficient mutation coverage, barcode diversity, and informative barcodes (Figure 1B)."**

**This seems to apply only to F13. In contrast, F43 suggests that ~150 transformants would suffice. This sizable difference deserves discussion. What accounts for the discrepancy? Were similar analyses performed for other regions? If so, do additional patterns emerge? I am also uncertain how many transformants per bp were finally used for the functional screening of Figure 2.**

Response:

Following the clarifications provided in the previous comment#3.3, the analysis indicates that the optimal library quality is achieved at **~**350 transformants per base pair for fragment F13 and **~**300 transformants per base pair for fragment F43. Although the F43 fragment showed a slightly lower number of transformants, it is important to note that these values remain of the same order of magnitude. This difference likely reflects fragment-specific factors, such as local sequence composition, synthesis variability within oPools, rather than a systematic bias in the method.

However, this type of analyses were done only on these two regions. To assure a robust mutant library, we then used the threshold of ~350 transformants per base pair (after Gibson cloning) to construct the entire *PDR1* plasmids library. All 43 fragment libraries exhibited high mutation coverage and barcode diversity, as reported in Supplementary Table 8 and Supplementary Figures 4 and 5.

For the functional screening shown in Figure 2, the F13 library was generated using ~25,000 transformants after Gibson assembly (~350 transformants per bp), ~100,000 transformants after Golden Gate assembly, and ~100,000 transformants following transformation into *S. cerevisiae*.

## **Comment #3.5. * Figures 1C-D:**

**I had not realized previously (though it now seems obvious) that the number of barcodes was clipped at 10. As Reviewer 1 suggested, the non-clipped versions are now shown in Sup. Figs. 1 and 2. In my opinion, these non-clipped figures should appear in the main text, as the clipped versions obscure important information. The observation that only a few mutations have fewer than 5 barcodes can instead be described in the text, with reference to the clipped data as supplementary information (or very low barcode counts could be color-coded differently in the main figure).**

Response:

As suggested, we have now included non-clipped versions of Figures 1C and 1D in the main text, in which very low barcode counts are highlighted using a distinct color scale. To preserve clear visualization of low barcode counts, the clipped versions (clipped at 10 barcodes) have been moved to the Supplementary Information (Supplementary Figures 1 and 2). Observations regarding mutations supported by fewer than four barcodes are now explicitly described in the main text, with references to the supplementary figures.

Revised Figure 1C and 1D:

**Library quality control after (C) Gibson and (D) Golden Gate assemblies for *PDR1* F13 and F43**. Heatmaps show barcode diversity for each possible amino acid substitution at each codon position. For each fragment, a total of 25,000 and 100,000 transformants were recovered and analyzed after Gibson and Golden Gate assemblies, respectively. Barcode diversity is shown using an unclipped color scale, allowing visualization of the full range of barcode counts. Mutations covered by high barcode diversity (#barcodes >=10 and >=4) are represented by blue and purple scales, respectively, while lower barcode diversity (#barcodes <4) is represented by a red scale. Grey squares represent WT amino acids. The numerical data underlying these graphs is provided in the files S2_Data.xlsx and S3_Data.xlsx. Clipped versions of these graphs (maximum of 10 barcodes per mutation), optimized to highlight lower barcode diversity, are provided in Supplementary Figures 1 and 2.

Revised Supplementary Figure 1:

**Supplementary Figure 1: Barcode diversity after Gibson assembly.** Heatmaps show the total number of unique barcodes for each possible amino acid substitution at each codon position in Pdr1 fragments F13 and F43. For each fragment, a total of 25,000 transformants were recovered and analyzed. Covered mutations are mutations represented by #barcodes >0, while barcode diversity represented by #barcodes >4 or >9 per mutation demonstrates an increasing number of replicates for a same mutation. Grey squares represent WT amino acids. In the heatmaps, the number of barcodes per mutation is clipped at 10. The numerical data underlying this graph is provided in the file S2_Data.xlsx

Revised Supplementary Figure 2:

**Supplementary Figure 2: Barcode diversity after Golden Gate assembly.** Heatmaps show the total number of unique barcodes for each possible amino acid substitution at each codon position in Pdr1 fragments F13 and F43. For each fragment, a total of 100,000 transformants were recovered and analyzed. Covered mutations are mutations represented by #barcodes >0, while barcode diversity represented by #barcodes >4 or >9 per mutation demonstrates an increasing number of replicates for a same mutation. Grey squares represent WT amino acids. In the heatmaps, the number of barcodes per mutation is clipped at 10. The numerical data underlying this graph is provided in the file S3_Data.xlsx.

Main text, *started line 215*:

“A random sampling of resulting distributions indicates that after Gibson assembly, ~350 transformants per bp are needed to optimize sufficient mutation coverage, barcode diversity, and informative barcode (Figure 1B). This level of recovery yields 300-350 informative barcodes covering all possible mutations, with over 99.8% of mutations represented by #barcodes >4 and over 97.0% by #barcodes >9 (Figure 1B, Supplementary Figure 1). Altogether, the findings demonstrate excellent mutation coverage and barcode diversity, after the first cloning step, two essential features for the robustness and reproducibility of DMS data.

As each cloning step inevitably results in some diversity loss, the number of transformants recovered after Golden Gate assembly was increased fourfold (~1400 transformants per bp). Although a small subset of mutations showed lower representation, our cloning strategy ensured over 99.8% of mutation coverage and preserved high barcode diversity: over 93.6% of mutations were still covered by #barcodes >4, and over 68.6% by #barcodes >9 ( Supplementary Figure 2).”

Methods, *started line 560*:

“The presence of all expected mutations, with excellent DNA barcode diversity per mutation was confirmed in libraries F13 and F43 (after Gibson assembly: all mutations are covered by #barcodes >4 and after Golden Gate assembly: ~93.6-100% of mutations are covered by #barcodes >4) (Figure 1C and D, Supplementary Figures 1 and 2). A small number of mutations, specifically those at residues 322 to 324, were detected at lower read counts. Nevertheless, they still showed high mutation coverage (98.3%) and acceptable barcode diversity, with 71.7% of mutations with #barcodes > 4 (Figure 1D, Supplementary Figure 2), which is the threshold required to have over 90% accuracy in selection coefficients (Figure 2C).”

## **Comment #3.6. The new Sup. Figs. 1 and 2 make it clear that systematic biases exist in barcode coverage. Much of this can be attributed to the number of synonymous codons generated by NNK mutagenesis. For example, Leucine, Arginine, and Serine have more barcodes because they are encoded by three possible codons. Valine, Alanine, Glycine, and Proline each have two codons, yet Proline and Alanine appear underrepresented in both F13 and F43. Since Sup. Figs. 7 and 8 are also clipped at 10, it is difficult to assess whether this pattern generalizes more broadly. I have no personal experience with NNK mutagenesis—perhaps these biases are known—but they merit some discussion, as they represent a minor limitation compared with more expensive strategies in which amino acid substitutions are preprogrammed in the oligo sequences.**

Response:

We agree that systematic biases in barcode diversity are observed and merit discussion. To better characterize these effects, we generated additional supplementary figures showing barcode diversity (i) at the codon level and (ii) using unclipped scales across the full *PDR1* library (Supplementary Figures 3-8). These analyses support three main points:

1. As noted by the reviewer, amino acids encoded by more synonymous NNK codons (e.g., leucine, arginine, serine) systematically show higher barcode diversity. This is an expected property of NNK mutagenesis and is now mentioned in the manuscript.
2. Codon-level analyses (Supplementary Figures 3 and 4) indicate that barcode diversity cannot be explained solely by the number of synonymous codons. Certain codons (e.g., G-rich or T-rich codons) are consistently overrepresented, while others (e.g., proline-, alanine-, or threonine-associated codons) are underrepresented despite similar codon multiplicity. This pattern is observed both in fragments F13/F43 and across the full gene, suggesting biases introduced during oligonucleotide pool synthesis.
3. We also observe clear position-specific effects, with a small number of codon positions showing markedly reduced or absent representation due to incomplete oPool synthesis. These effects are visible both locally (F13: positions 301 and 322-224 are less represented) and across the entire *PDR1* library (19 positions are absent from the library - shown in bright red in Supplementary Figures 5-8).

Overall, these results indicate that biases related to barcode diversity are mainly due to (i) properties of NNK mutation and (ii) technical limitations of oligonucleotide pool synthesis. We now address these biases in the manuscript.

Revised Supplementary Figure 3:

**Supplementary Figure 3: Barcode diversity after Gibson assembly.** Heatmaps show barcode diversity for each possible NNK codon substitution at each codon position in Pdr1 fragments F13 and F43. For each fragment, a total of 25,000 transformants were recovered and analyzed. Barcode diversity is shown using an unclipped color scale, allowing visualization of the full range of barcode counts. Mutations covered by high barcode diversity (#barcodes >=10 and >=4) are represented by a blue and purple scale, respectively, while lower barcode diversity (#barcodes <4) is represented by a red scale. Grey squares represent WT amino acids. The numerical data underlying this graph is provided in the file S2_Data.xlsx.

Revised Supplementary Figure 4:

**Supplementary Figure 4: Barcode diversity after Gibson assembly.** Heatmaps show barcode diversity for each possible NNK codon substitution at each codon position in Pdr1 fragments F13 and F43. For each fragment, a total of 100,000 transformants were recovered and analyzed. Barcode diversity is shown using an unclipped color scale, allowing visualization of the full range of barcode counts. Mutations covered by high barcode diversity (#barcodes >=10 and >=4) are represented by a blue and purple scale, respectively, while lower barcode diversity (#barcodes <4) is represented by a red scale. Grey squares represent WT amino acids. The numerical data underlying this graph is provided in the file S3_Data.xlsx.

Revised Supplementary Figure 5:

**Supplementary Figure 5: Library quality control per amino acid substitution after Gibson assembly of the full-length *PDR1* sequence (43 fragments).** Heatmap displays the barcode diversity associated with each possible amino acid substitution at each codon position. Barcode diversity is shown using an unclipped color scale, allowing visualization of the full range of barcode counts. Mutations covered by high barcode diversity (#barcodes >=10 and >=4) are represented by a blue and purple scale, respectively, while lower barcode diversity (#barcodes <4) is represented by a red scale. Grey squares represent WT amino acids. The numerical data underlying this graph is provided in the file S2_Data.xlsx.

Revised Supplementary Figure 6:

**Supplementary Figure 6: Library quality control per amino acid substitution after Golden Gate assembly of the full-length *PDR1* sequence (43 fragments).** Heatmap displays the barcode diversity associated with each possible amino acid substitution at each codon position. Barcode diversity is shown using an unclipped color scale, allowing visualization of the full range of barcode counts, including low values in red. Mutations covered by high barcode diversity (#barcodes >=10 and >=4) are represented by a blue and purple scale, respectively, while lower barcode diversity (#barcodes <4) is represented by a red scale. Grey squares represent WT amino acids. The numerical data underlying this graph is provided in the file S3_Data.xlsx.

Revised Supplementary Figure 7:

**Supplementary Figure 7: Library quality control per NNK codon substitution after Gibson assembly of the full-length *PDR1* sequence (43 fragments).** Heatmaps show barcode diversity for each possible NNK codon substitution at each codon position. Barcode diversity is shown using an unclipped color scale, allowing visualization of the full range of barcode counts. Mutations covered by high barcode diversity (#barcodes >=10 and >=4) are represented by a blue and purple scale, respectively, while lower barcode diversity (#barcodes <4) is represented by a red scale. Grey squares represent WT amino acids.The numerical data underlying this graph is provided in the file S2_Data.xlsx.

Revised Supplementary Figure 8:

**Supplementary Figure 8: Library quality control per NNK codon substitution after Golden Gate assembly of the full-length *PDR1* sequence (43 fragments).** Heatmaps show barcode diversity for each possible NNK codon substitution at each codon position. Barcode diversity is shown using an unclipped color scale, allowing visualization of the full range of barcode counts, including low values in red. Mutations covered by high barcode diversity (#barcodes >=10 and >=4) are represented by a blue and purple scale, respectively, while lower barcode diversity (#barcodes <4) is represented by a red scale. Grey squares represent WT amino acids. The numerical data underlying this graph is provided in the file S3_Data.xlsx.

Main text, *started line 573*:

“Analysis of the complete 43-fragment library confirmed strong performance across all quality metrics. Nearly all expected mutations (∼98,4%) were recovered, with only a few absent positions likely attributable to synthesis dropout in the oPools. Barcode diversity per mutation remained high throughout all libraries: after Gibson assembly, ~97,5% mutations were represented by #barcodes >4, and after Golden Gate assembly this remained true for ~95.4% of positions (Supplementary Figures 5-8; Supplementary Table 8). Uniformity analysis further indicated that variant abundance across the library (Gini coefficient ~0.32) was well balanced and acceptable for downstream DMS experiments (Supplementary Table 8).

Consistent with previous observations using NNK degenerate codon libraries, expected biases in variant diversity were observed across amino acid substitutions as some amino acids are encoded by more codons. Amino acids such as leucine, arginine, and serine are encoded by three codons, while most others are encoded by one or two, resulting in higher expected representation for these residues (Figure 1C and D, Supplementary Figures 5 and 6).

However, codon-level analyses revealed that synonymous codon multiplicity alone does not fully explain the observed patterns (Supplementary Figures 3, 4, 7 and 8). Certain codons rich in thymine or guanine (e.g., TTT, GGG, TGG, TTG) consistently showed higher barcode diversity, while others were underrepresented, such as the codons for proline, threonine, and alanine (Supplementary Figures 3-8). In addition, position-specific biases were detected along the gene, with a small number of codon positions showing reduced (*PDR1* F13 - codons 301 and 322-324: Supplementary Figures 3 and 4) or absent representation (19 codon positions in the entire *PDR1* gene: Supplementary Figures 5-8), likely reflecting dropouts during oPool synthesis rather than cloning inefficiencies.

We additionally quantified the representation of non-programmed variants and found that ~10% of reads corresponded to the WT sequence and ~10% to double-mutants (Supplementary Table 8). These species can either be excluded during downstream fitness estimation or leveraged for dedicated analyses of epistatic interactions, depending on the experimental aims.”

**Comments from the Academic Editor:**

## **Comment #4.1. The new Figure 2C is almost unreadable; at least the text size has to be increased.**

Response:

We have increased the text size throughout Figure 2C, with particular attention to the axis titles and labels, to improve readability.

## **Comment #4.2. All code should be archived (e.g., on Zenodo) - the github links should be replaced with dois to the archived versions.**

Response:

We agree. We have archived the current version of our publicly available GitHub repository on Zenodo, which generated the following DOI: 10.5281/zenodo.18342596. This DOI has now been included in the Data Accessibility Statement, together with the GitHub repository link.

**Comment #4.3. In Figure 1 C and D it should say ">=10" in the heatmap legend.**

Response:

As suggested, we have updated the heatmap legends in Figures 1C and 1D to explicitly indicate “>=10”.

**Comment #4.4. In the Supplement, I would have appreciated an actual distribution plot of the barcode numbers in addition to the heatmaps and the uniformity coefficients.**

Response:

As suggested, we have added a new supplementary figure (Supplementary Figure 11) showing the full distribution of the number of barcodes per amino acid mutation for fragments F13 and F43 after both Gibson and Golden Gate assemblies. This distribution plot complements the heatmaps and uniformity metrics by providing a direct view of barcode count variability across mutations.

Revised Supplementary Figure 11:

**Supplementary figure 9. Distribution of barcode counts per amino‑acid mutation after (A) Gibson and (B) Golden Gate assemblies for Pdr1 F13 and F43.** Histograms show the number of unique barcodes associated with each amino‑acid substitution in fragments F13 (gray) and F43 (blue). For each fragment, the x‑axis indicates the number of barcodes linked to a given amino‑acid substitution, and the y‑axis shows the number of substitutions observed at each barcode count. The numerical data underlying these graphs is provided in the files S2_Data.xlsx and S3_Data.xlsx.

Main text, *started line 229*:

“To complement the barcode diversity and coverage analyses, the full distributions of barcode counts per amino acid mutation after Gibson and Golden Gate assemblies are shown in Supplementary Figure 9, providing a direct visualization of barcode representation across variants for fragments F13 and F43. In addition, the uniformity of variant representation was assessed after both the Gibson and the Golden Gate steps using the Gini coefficient, an index of distribution bias ranging from 0 (perfect uniformity) to 1 (maximum bias) [[31]](https://paperpile.com/c/yWCLMT/pkxmM). We observed that the Golden Gate assemblies showed a slight increase in the Gini coefficient compared to the Gibson assemblies (Supplementary Figures 10 and 11), reflecting a minimal bottleneck related to multi-fragment cloning. Overall, uniformity remained high across all 43 fragments (Gini coefficients consistently <0.5).”
